# Cranberry Proanthocyanidins and Dietary Oligosaccharides Synergistically Modulate *Lactobacillus plantarum* Physiology

**DOI:** 10.3390/microorganisms9030656

**Published:** 2021-03-22

**Authors:** Ezgi Özcan, Michelle R. Rozycki, David A. Sela

**Affiliations:** 1 Department of Food Science, University of Massachusetts, Amherst, MA 01003, USA; eozcan@umass.edu (E.Ö.); mrozycki@umass.edu (M.R.R.); 2 Department of Microbiology & Physiological Systems, University of Massachusetts Medical School, Worcester, MA 01003, USA

**Keywords:** polyphenols, probiotics, lactobacillus, oligosaccharides, nutrition, cranberry

## Abstract

Plant-based foods contain bioactive compounds such as polyphenols that resist digestion and potentially benefit the host through interactions with their resident microbiota. Based on previous observations, we hypothesized that the probiotic *Lactobacillus plantarum* interacts with cranberry polyphenols and dietary oligosaccharides to synergistically impact its physiology. In this study, *L. plantarum* ATCC BAA-793 was grown on dietary oligosaccharides, including cranberry xyloglucans, fructooligosaccharides, and human milk oligosaccharides, in conjunction with proanthocyanidins (PACs) extracted from cranberries. As a result, *L. plantarum* exhibits a differential physiological response to cranberry PACs dependent on the carbohydrate source and polyphenol fraction introduced. Of the two PAC extracts evaluated, the PAC1 fraction contains higher concentrations of PACs and increased growth regardless of the oligosaccharide, whereas PAC2 positively modulates its growth during xyloglucan metabolism. Interestingly, fructooligosaccharides (FOS) are efficiently utilized in the presence of PAC1, as this *L. plantarum* strain does not utilize this substrate typically. Relative to glucose, oligosaccharide metabolism increases the ratio of secreted acetic acid to lactic acid. The PAC2 fraction differentially increases this ratio during cranberry xyloglucan fermentation compared with PAC1. The global transcriptome links the expression of putative polyphenol degradation genes and networks and metabolic phenotypes.

## 1. Introduction

Polyphenols are bioactive antioxidant molecules with epidemiological and mechanistic evidence for their anti-inflammatory, anticancer, and antimicrobial effects in humans and other mammals [1,2]. Polyphenols are acquired primarily from dietary plant materials with their bioavailability largely dependent on structural modifications catalyzed by gut microbiota, including decarboxylation, deglycosylation, and demethylation, among other activities [3]. Cranberries, in particular, are enriched in several polyphenol classes, including proanthocyanidins (PACs), anthocyanidins, flavanols, and benzoic acids [4]. PACs are oligomeric flavonoids comprised mainly of catechin and/or epicatechin units [5]. The cranberry cell wall incorporates mostly A-type PACs, which contribute a significant fraction of the total flavanols on a weight basis [4]. Cranberries are often used in polyphenol-enriched food products that have been reported to be effective in addressing obesity, inflammation, and cardiovascular disease [6,7]. Specifically, A-type PACs are linked with inhibiting the adhesion of uropathogenic *Escherichia coli*, which is an essential step in the development of urinary tract infections [8]. Lower molecular weight metabolites exhibit increased bioavailability in addition to their antioxidant potential.

Lactic acid bacteria (LAB) interact with polyphenols in native and agricultural plants and do so in various vegetable and fruit fermentations [9]. In addition, LAB species are commensals of the human gut microbiome, and selected strains are used as probiotics [10]. Specifically, *Lactobacillus plantarum* (recently reclassified as *Lactiplantibacillus plantarum* [11]) is a popular probiotic candidate due to the ease of working with it, human origin, and ability to survive gastrointestinal transit. Moreover, *L. plantarum* has been extensively researched in addressing disease states, maintaining gut homeostasis, and as a platform to deliver vaccines [12]. There is evidence for polyphenols enriching specific gut microorganisms through promoting carbohydrate uptake and potentially providing an energy source for cellular growth analogous to the prebiotic effect [13], although the latter is currently a matter of active scientific debate and inquiry. It is generally accepted, however, that polyphenols could act as antimicrobials against certain pathogens and, potentially, against commensals [14]. This altogether makes the combination of LAB strains with prebiotics and polyphenols good candidates for a “synergistic synbiotic” with the intention to interact specifically with the co-administered microorganisms [15].

Dietary plant materials contain fibers that are often resistant to digestion and, thus, fermented by resident microbes within the colon. Dietary fibers include polysaccharides such as pectins, xylans, or oligosaccharides that vary by constituent monosaccharide residues and degree of polymerization (DP). Cranberry fruit contains soluble dietary fibers that may function as a prebiotic through the selective enrichment of beneficial microbes [16]. Accordingly, cranberry hull extract contains two arabinoxyloglucan species, heptasaccharides, and octasaccharides [17]. Furthermore, cranberry xyloglucans are β-(1-4)-linked D-(+)-glucopyranose that may be mainly substituted with an α-linked D-(+)-xylopyranosyl side chain and α-linked L-(+)-arabinofuranosyl unit. Additional side chains substituents have been identified with galactose, rhamnose, galacturonic acid, and fucose [17]. Cranberry products often contain pectic oligosaccharides resulting from pectinase hydrolysis. In general, cranberry extracts contain a complex mixture of both pectic (e.g., homogalacturonan) and xyloglucan oligosaccharides, as well as other carbohydrates such as arabinans [17]. Interestingly, soluble xyloglucans shift the gut microbiome composition to a greater extent than pectic polymers during in vitro fermentation [18].

Plant polyphenols are distributed as soluble molecules in vacuoles and insoluble polyphenols bound within the cell wall. The latter are often challenging to separate from dietary fibers (i.e., polysaccharides) in industrial processes [19]. This is due to hydrophobic aromatic rings and/or hydrophilic hydroxyl groups linking the polyphenols to fiber. Thus, dietary fibers may carry polyphenols during intestinal transit, where they could be released by gut microbiota activity [20]. In cranberries, PACs are both soluble and insoluble and remain associated with fiber and protein after extraction [21]. Given their association within foods, as well as throughout digestion, gut microbes interact simultaneously with cranberry fiber and polyphenols.

We previously reported that the *Bifidobacterium longum* and *L. plantarum* strains utilize cranberry xyloglucans to accumulate a biomass and produce formic acid, among other fermentative end-products. In addition, contaminant phytochemicals (e.g., polyphenols) in crude xyloglucan extracts enhance bacterial growth [16]. This formed the hypothesis that cranberry polyphenols and oligosaccharides interact synergistically with commensal LAB, potentially through increasing the cellular capacity to obtain and metabolize carbohydrates. In order to test this, the differential physiology was evaluated in *L. plantarum* ATCC BAA-793 exposed to combinations of cranberry PACs with dietary oligosaccharides as the sole carbohydrate source.

## 2. Materials and Methods

### 2.1. Bacterial Propagation and Growth Phenotype Assay

Bacterial cells were routinely propagated in de Man, Rogosa, and Sharpe (MRS; BD Difco, Franklin Lakes, NJ, USA) medium supplemented with 0.05% (*w*/*v*) L-cysteine hydrochloride (Across Organics, Fair Lawn, NJ, USA) at 37 °C under anaerobic conditions (Coy Laboratory Products, Grass Lake, MI, USA). Bacterial growth was evaluated in a 96-well microplate reader anaerobically at 37 °C for 48 h by assessing the optical density at 600 nm (OD_600nm_) in an automated PowerWave HT microplate spectrophotometer (BioTek Instruments, Vinooski, VT, USA). Overnight cultures were inoculated (1%, *v*/*v*) into experimental media based on a modified MRS media formulation without an acetate and carbohydrate source (mMRS). Sole carbohydrate sources were added at a final concentration of 1% (*w*/*v*). This includes glucose (Sigma-Aldrich Co., St. Loius, MO, USA), cranberry oligosaccharides (donated by Ocean Spray Inc, Lakeville-Middleboro, MA, USA and extracted as described previously [22]), fructooligosaccharides (FOS) from chicory (≥90%, DP ≥ 10, Sigma-Aldrich Co., St. Loius, MO, USA), and HMO (donated by Daniela Barile, UC Davis, CA, USA). Pooled HMO was extracted as described previously [23] and contained a total of 36.6% (*w/w*) HMO with DP≤5 (3’fucosyllactose (FL), 2’FL, lacto-N-tetraose (LNT), lacto-N-neotetraose (LNnT), lacto-N-fucopentaose (LNFP) I, 3’sialyllactose (SL), and 6’SL), 1 % (*w/w*) monomers, and 53.6% (*w/w*) uncharacterized HMO. PACs (donated by Ocean Spray Inc.) were isolated as described previously [24] (the composition is reported in Table S1) and were dissolved in dimethyl sulfoxide (DMSO, Fisher Chemicals, Hampton NH, USA) to be diluted in mMRS at a final concentration of 1 mg/mL. Each experiment was evaluated in biological triplicates with technical triplicates. Negative controls consisted of inoculated medium in the absence of carbohydrates and PACs.

Growth characteristics were determined using the Growthcurver package in R [25], with the resultant kinetic data was subjected to a one-way ANOVA with Tukey’s honestly significant difference (HSD) test for multiple comparisons. Bacterial strains that were not indicated as American Type Culture Collection (ATCC, Manassas, VA, USA) were acquired from the USDA Agricultural Research Service Culture Collection (NRRL, Peori, IL, USA).

### 2.2. Bacterial Metabolic End-Product Quantification

Cell-free supernatants were harvested from the microplate at the stationary phase and filtered through a 0.22-µm membrane (Costar Spin-X, Corning, Glendale, AR, USA) following centrifugation and stored at −20 °C. Secreted bacterial metabolites were quantified using an Agilent 1260 Infinity High Performance Liquid Chromatography (HPLC) system (Agilent Technologies, Santa Clara, CA, USA) equipped with a Wyatt Optilab T-rEX refractive-index detector (Wyatt Technology Corp., Santa Barbara, CA, USA). Separation was performed on an Aminex HPX-87H column (7.8 mm ID × 300 mm, Bio Rad Laboratories, Hercules, CA, USA) at 30 °C in a mobile phase of 5 mM H_2_SO_4_ at a flow rate of 0.6 mL/min, with an injection volume of 20 µL. Organic acid (acetic, lactic, and formic acids) standards were acquired from Sigma-Aldrich Co. (St. Louis, MO, USA). Metabolite concentrations were calculated from standard curves generated from external standards for six concentrations (0.5, 1, 5, 10, 20, and 50 mM). Quantification was performed in biological triplicates with each HPLC measurement performed in duplicate. The resultant metabolites data was subjected to one-way ANOVA with Tukey’s HSD test for multiple comparisons using GraphPad Prism, version 8 (GraphPad Software Inc., San Diego, CA, USA).

### 2.3. PAC Degradation Product Identification

Ultra-performance liquid chromatography coupled to a Xevo TQD triple quadrupole mass spectrometer (Waters Corp., Milford, MA, USA) was used to identify the phenolic metabolites following *L. plantarum* fermentations with PACs. Separation was conducted as previously reported [26] and performed on a C18 column (100 × 1.0 mm ID; Waters Corp., Milford, MA, USA) at 40 °C with an aqueous phase (2% acetic acid (*v*/*v*)) and an organic phase (2% acetic acid in acetonitrile (*v*/*v*)) at a flow rate of 0.5 mL/min. The total run time was 18 min, with the organic phase applied to the gradient as: 0 min, 0.1%; 1.5 min, 0.1%; 11.17 min, 16.3%; 11.5 min, 18.4%; 14 min, 18.4%; 14.1 min, 99.9%; 15.5 min, 99.9%; and 15.6 min, 0.1%. UV measurements were taken at λ = 280 nm. Electrospray ionization was operated in negative mode with the following parameters: capillary voltage, 3 kV; source temperature, 130 °C; desolvation temperature, 400 °C; desolvation gas (N_2_) flow rate, 750 L/h; and cone gas (N_2_) flow rate, 60 L/h. MS/MS parameters (cone voltage and collision energy) were optimized by direct infusion using 10 μg/mL solutions at a flow rate of 5 μL/min. The MS/MS conditions for each standard are provided in Table S2. Three groups of standards were prepared for separating their elution times and mass spectra using the multiple reaction monitoring (MRM) mode. Data acquisition and processing was performed by MassLynx 4.1 software (Waters Corp., Milford, MA, USA).

### 2.4. RNA-seq Transcriptome Library Preparation

Three milliliters of cells were harvested by centrifugation at the mid-exponential phase and resuspended in 500 μL RNAlater. The samples were incubated at 4 °C overnight and transferred to −80 °C for storage. The cells were pelleted through centrifugation and washed twice with phosphate buffer to remove the residual RNAlater. Total RNA was extracted using the Ambion RNAqeous kit (Invitrogen Corp., Carlsbad, CA, USA) according to the protocol, with minor modifications. Cells were suspended in lysis buffer in Lysing Matrix E bead beating tubes to disrupt cell walls with a FastPrep 24 bead beater (MP Biomedicals, Santa Ana, CA, USA). Bead beating was performed twice at 5.5 m/s for 30 s with 1 min incubation on ice. The RNAqeous protocol was followed and terminated with total RNA elution into 50 µL of elution solution.

Total RNA concentrations were measured on a NanoDrop spectrophotometer (Thermo Scientific, Waltham, MA, USA), and genomic DNA was removed with the Ambion Turbo DNA-free DNase kit (Invitrogen Corp., Carlsbad, CA, USA) for 30 min at 37 °C. Residual genomic DNA contamination was evaluated by qRT-PCR using the PowerUp SYBR Green kit (Applied Biosystems, Foster City, CA, USA) with primers targeting the *L. plantarum rpoD* gene, as described previously [27]. DNase treatment was repeated for a second time when necessary. DNA-free RNA was quantified using the Qubit High-Sensitivity (HS) RNA Assay Kit (Invitrogen Corp., Carlsbad, CA, USA), and RNA integrity was tested using High-Sensitivity RNA screen tapes and reagents on a TapeStation 2200 system (Agilent Technologies, Santa Clara, CA, USA). Samples exhibiting an RNA Integrity Number equivalent (RIN^e^) greater than 7.0 were subjected to ribosomal RNA depletion via the Ribo-Zero Magnetic Kit for bacteria (Illumina Inc., San Diego, CA, USA). Subsequently, mRNA purification was performed using the RNeasy MinElute Cleanup Kit (Qiagen, Hilden, Germany). mRNA concentrations were quantified with the Qubit HS RNA Assay, and rRNA depletion was confirmed through the absence of 16S and 23S peaks on the TapeStation 2200 system.

mRNA-enriched samples with RIN^e^ scores ~ 1.0 were selected for sequencing library preparation with the NEBNext Ultra II Directional kit (New England Biolabs Inc., Ipswich, MA, USA). The purification steps adhered to kit instructions for rRNA depleted RNA using AMPure XP beads (Beckman Coulter, Inc., Brea, CA, USA) and indexed using NEBNext Multiplex Oligos for Illumina (Dual Index Primers Set; New England Biolabs Inc., USA). First- and second-strand complementary DNA (cDNA) synthesis were performed following the library generation protocol. Preparative PCR cycles ranged from 8–12 according to the input RNA concentration. Libraries were quantified with the Qubit double-stranded DNA HS assay (Invitrogen Corp., Carlsbad, CA, USA). The library quality was assessed through a DNA analysis ScreenTape assay on the TapeStation 2200 system. The libraries were pooled in equimolar concentration (0.5 nM) and denatured immediately prior to sequencing following standard Illumina procedures. Sequencing was performed on an Illumina NextSeq platform (paired-end, 2 × 75 bp, 5% Phi-X).

### 2.5. Bioinformatic and Statistical Analysis of Transcriptome Data

Sequencing reads were uploaded to the Massachusetts Green High-Performance Computing Cluster for downstream bioinformatic and statistical analyses, unless specifically noted. Sequencing adaptors were trimmed using Trimmomatic (v.0.32) [28]. The reads were aligned to the 16S rRNA gene region obtained from Silva bacterial reads (https://www.arb-silva.de/, accessed on 29 January 2019) using Bowtie2 (v2.3.4.3) [29]. Samples exhibiting an alignment rate to the 16S gene exceeding 1% were removed from 16S contamination using bbduk in bbmap (v38.34) [30]. The resultant reads were aligned to the reference genome (NCBI accession number: AL935263) using Bowtie2 [29]. Aligned reads were then name-sorted using Samtools (v1.4.1) [31], and the total and specific gene counts were obtained by overlapping of a specific genomic locus (i.e., locus tag) with genomic feature and annotation (GFF) using HTSeq (v0.10.0) [32].

### 2.6. Differential Gene Expression Analysis

In order to identify and quantify the magnitude by which genes are differentially expressed, the R package DESeq2 (v1.26.0) was used to analyze the raw count data [33]. DESeq2 applied the Wald test for statistical analysis, and adjusted *p*-values ≤ 0.05 were defined as significant. A principal component analysis was conducted and visualized by DESeq2. Volcano plots were visualized using EnhancedVolcano [34]. Gene expression was calculated from regularized log-transformed counts, ranked by log_2_ fold change, and visualized in a heatmap.

### 2.7. Transcriptome Functional Annotation and Enrichment Analysis

Genes were ranked by the log_2_ fold change of pair-wise comparisons, without independent filtering for the gene ontology (GO) enrichment analysis performed by R package goseq (v1.38.0) [35]. GO annotations and IDs were obtained from UniProt and used for GO mapping. The gene feature FASTA file was used for length-based biases. Differences with *p*-values ≤ 0.05 were considered significant. The R package ggplot2 (v3.3.0) [36] was used to generate the bubble graph to visualize the GO enrichment.

## 3. Results

### 3.1. Cranberry PACs Increase Biomass Accumulation during L. plantarum Dietary Oligosaccharide Fermentation

PACs isolated from cranberries are enriched in proanthocyanidins, with the composition of the two fractions reported in Table S1. The PAC1 fraction was obtained from acetone extraction, and PAC2 was the concentrate passed through a resin column to enrich for proanthocyanidins. Both fractions were spray-dried and dissolved in DMSO prior to experiments. The PAC1 fraction contained higher concentrations of PACs and was lower in total anthocyanidins, phenolic acids, sugars, and organic acids. PAC2 contained 0.8% maltodextrin on a dry basis, although *L. plantarum* ATCC BAA-793 did not utilize this substrate in vitro (data not shown).

**Figure 1:**
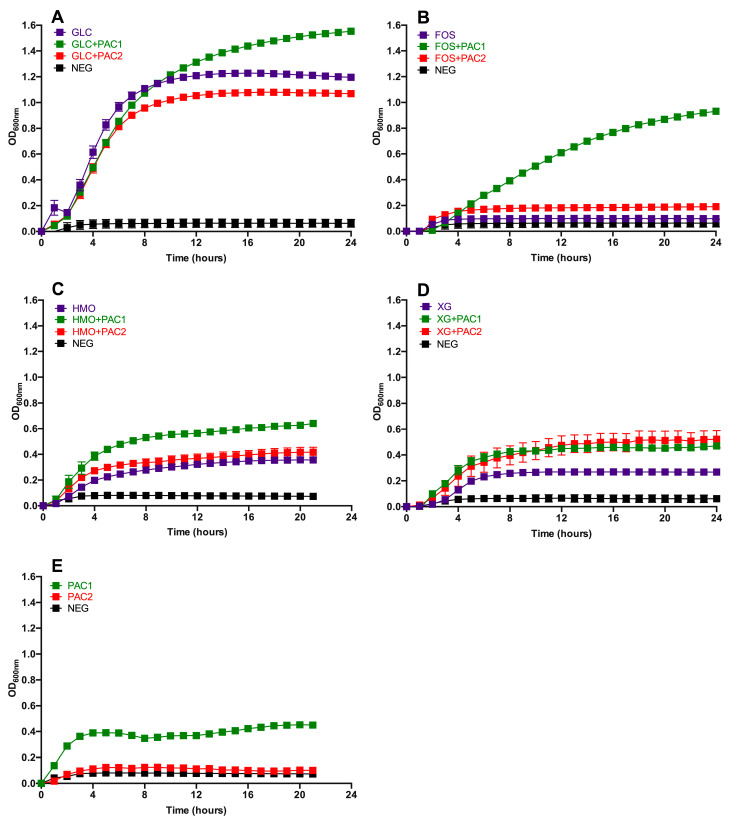
*Lactobacillus plantarum* growth on oligosaccharides in the presence of cranberry proanthocyanidins (PACs). Growth curves of *Lactobacillus plantarum* ATCC BAA-793 on modified de Man, Rogosa, and Sharpe (MRS) containing 1-mg/mL PACs, along with 1% (*w*/*v*) glucose (GLC) (**A**), fructooligosaccharides (FOS) (**B**), human milk oligosaccharides (HMO) (**C**), xyloglucans (XG) (**D**), and PACs alone (**E**). The curves are drawn from the average of three independent biological replicates. NEG (negative control) is modified MRS without a carbohydrate source.

Several concentrations of PACs were tested in conjunction with glucose and did not exert a significant impact on *L. plantarum* growth; thus, 1.0 mg/mL was selected for the subsequent experiments (Figure S1). The DMSO concentration used to dissolve PAC extracts did not hinder *L. plantarum* growth as well (data not shown). The *L. plantarum* growth while fermenting glucose, FOS, HMO, and xyloglucans (XG) in combination with PACs is depicted in Figure 1, with the growth kinetics reported in Table S3. During glucose fermentation, PAC1 enhanced *L. plantarum* growth relative to glucose alone (OD_600nm_ = 1.48 ± 0.02 vs. 1.21 ± 0.03) and to a greater extent than the combination of glucose and PAC2 (OD_600nm_ = 1.07 ± 0.01; *p* < 0.05; Figure 1A). The growth rate on glucose remained constant regardless of the PAC fraction introduced to the medium (*p* > 0.05; Table S3).

Under the experimental conditions tested, *L. plantarum* ATCC BAA-793 did not grow on FOS as a sole fermentable substrate. Remarkably, and somewhat unexpectedly, the PAC1 fraction activated a FOS utilization phenotype in ATCC BAA-793 (OD_600nm_ = 1.02 ± 0.01; Figure 1B). PAC2 did so to a significantly lesser extent with respect to the biomass accumulation (OD_600nm_ = 0.25 ± 0.04) and growth rate (*p* < 0.05). Clearly, the cranberry PAC extract modified the *L. plantarum* physiology to utilize what was previously an unusable oligosaccharide substrate.

Interestingly, *L. plantarum* ATCC BAA-793 utilized HMO as a sole carbohydrate source, a previously uncharacterized phenotype for this species. PAC1 significantly increased the biomass in HMO fermentation, whereas PAC2 did not promote the same effect (*p* < 0.05; Figure 1C). Both PAC1 and PAC2 increased the *L. plantarum* growth on XG (*p* < 0.05; Figure 1D). In general, the fermentations were more efficient in the presence of PAC1, with the most pronounced impact observed during FOS utilization. The PAC fractions did not impact the growth rates, with the exception of PAC2 during XG fermentation (Table S3).

Interestingly, PAC1 enabled biomass accumulation, whereas PAC2 did not when introduced as the sole carbon source (Figure 1E). This was somewhat unexpected, given the higher total sugar content in PAC2, although it is relatively minimal in absolute concentrations. It is possible that the higher organic acid and phenolic acid contents in PAC2 may hinder growth. In addition, the PAC1 fraction may contain more proanthocyanidins bound to dietary fiber.

### 3.2. Cranberry Polyphenols Alter Oligosaccharide Utilization Phenotypes of Lactobacilli Strains

Probiotic or commensal strains were evaluated, in addition to *L. plantarum* ATCC BAA-793, for comparison. Here, the aim was to examine the strain- and species-specific phenotypes in response to PACs. The growth of lactobacilli on glucose, HMO, and XG are reported in Figure S2. Accordingly, *Lactobacillus johnsonii* ATCC 33200 and *Lactobacillus plantarum* ATCC 14917 exhibit significant phenotypic responses to PACs. PAC1 enhanced *L. johnsonii* growth on glucose (OD_600nm_ = 0.72 ± 0.01), XG (OD_600nm_ = 0.48 ± 0.02), and HMO (OD_600nm_ = 0.21 ± 0.00) compared to the corresponding carbohydrate source alone (*p* < 0.05; Figure S2E–G and Table S4). In contrast, PAC2 significantly increased *L. johnsonii* growth only on HMO (OD_600nm_ = 0.22 ± 0.02; *p* < 0.05). Similarly, PAC2 increased the *L. plantarum* ATCC 14917 HMO utilization efficiency (OD_600nm_ = 0.49 ± 0.04; *p* < 0.05), whereas PAC1 promoted growth on XG (OD_600nm_ = 0.50 ± 0.01; *p* < 0.05; Figure S2B,C and Table S4).

PACs did not significantly influence *Lactobacillus reuteri* B-14171 growth on glucose, XG, and HMO (*p* > 0.05; Figure S2M–O). PAC1 decreased *Lactobacillus pentosus* B-227 growth on glucose (*p* < 0.05), whereas PAC2 did not exhibit a discernable impact (Figure S2I and Table S4). PACs did influence the *L. pentosus* fermentation of HMO and XG (Figure S2J,K and Table S4).

### 3.3. Cranberry PACs Alter Secreted Metabolic End-Products during Oligosaccharide Utilization

**Figure 2:**
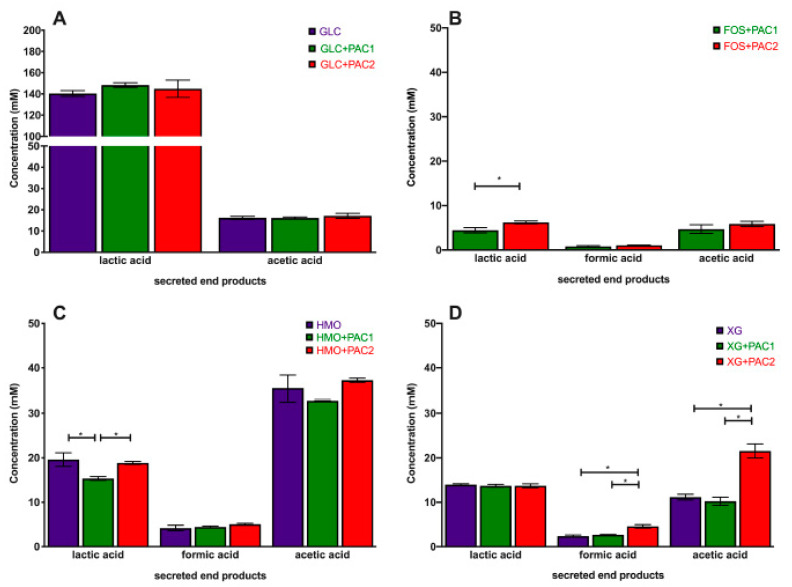
*Lactobacillus plantarum* secreted fermentative end-products while utilizing oligosaccharides and cranberry PACs. Absolute concentrations of lactic acid, acetic acid, and formic acid following the fermentation of glucose (**A**), fructooligosaccharides (**B**), human milk oligosaccharides (**C**), and xyloglucans (**D**). Averages from independent biological triplicates are depicted, and bars represent standard deviations of the mean. The values for organic acid production are expressed in millimolar absolute concentrations. Asterisks represent significant differences evaluated by two-way ANOVA and Tukey’s multiple comparisons test (*p* < 0.05).

The obligate heterofermentative *L. plantarum* utilizes glucose to secrete high concentrations of lactic acid (140.4 ± 2.7 mM) and acetic acid to a lesser extent (16.2 ± 0.7 mM; Figure 2A). *L. plantarum* strains generally secrete 20–50-fold more lactic acid than acetic acid during glucose fermentation [16,37,38]. Neither PAC fraction impacted the end-product profile during glucose fermentation. This includes formic acid, which was not detected during glucose fermentations, regardless of the PAC addition.

**Figure 3:**
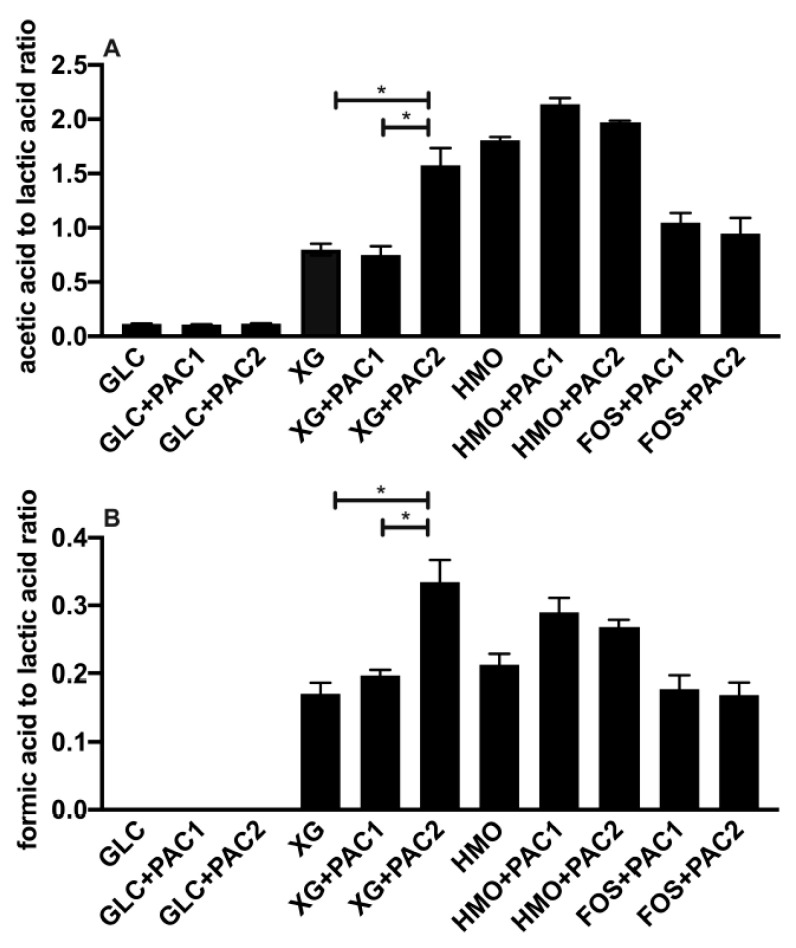
The ratio of fermentative end-products while utilizing oligosaccharides and cranberry PACs. Averages from independent biological triplicates with standard deviation of the mean of the acetic acid-to-lactic acid ratio (**A**) and formic acid-to-lactic acid ratio (**B**). Asterisks represent the significant difference evaluated by ANOVA and Tukey’s multiple comparisons test (*p* < 0.05).

During oligosaccharide fermentation, secreted acetic acid concentrations were higher relative to lactic acid, irrespective of the PACs. This was previously observed during *L. plantarum* arabinoxylo-oligosaccharide fermentation [38]. Figure 2 depicts the absolute concentrations of the end-products secreted into culture media, and Figure 3 reports the ratios of end-products to normalize the influence of the biomass variation. FOS utilization only occurs in the presence of PACs (Figure 2B), and the acetic acid-to-lactic acid ratio (AA:LA) increased appreciably relative to the AA:LA ratio during glucose fermentation (*p* < 0.05; Figure 3A). Thus, during FOS fermentation, acetic acid approaches equimolar concentrations with lactic acid, with the ratio approximately an order of magnitude larger than with glucose. It is notable that the end-product ratios remained constant regardless of the PAC fraction. This is despite PAC1 inducing dramatically more biomass accumulation during FOS fermentation, and the absolute concentrations of organic acids were very similar to those during growths with PACs alone (Figure S3).

HMO fermentation yielded AA:LA > 1.8, regardless of the PAC or its absence. Shifts in secreted end-products occurred generally in oligosaccharide fermentations with lower growth rates [16]. It is hypothesized that increased acetic acid secretion may be due, in part, to the hydrolysis of acetyl moieties within N-acetylglucosamine and N-acetylneuraminic acid residues. Similar observations were reported during the *L. plantarum* utilization of N-acetylglucosamine [37] and with other species utilizing HMO [39]. *L. plantarum* cranberry XG utilization favored acetic acid production as well, albeit to a lesser degree than FOS and HMO. The exception was *L. plantarum* XG fermentation in the presence of PAC2. In this instance, the ratio was ~1.5, which was similar to HMO utilization, although the XG oligosaccharides did not incorporate the acetyl groups (Figure 3A).

Invariably, *L. plantarum* secretes formic acid during oligosaccharide fermentation, including cranberry XG, as we previously reported [16]. The addition of PAC2 to XG fermentations induce formic acid production in absolute concentrations (Figure 2D) and relative to lactic acid (Figure 3B). This is consistent with the extreme skew towards acetic acid in the presence of PAC2. This suggests that the two-carbon acetic acid and one-carbon formic acid are being produced stoichiometrically at the expense of the three-carbon lactic acid. We previously observed similar phenomena during *Bifidobacterium longum* and *L. plantarum* xyloglucan fermentation without PACs [16]. It is not clear how PAC2 increases the relative acetic acid and formic acid concentrations during XG fermentation and if this signifies a link between the two cranberry products.

### 3.4. Biotransformation of Cranberry Phenolics Vary by Oligosaccharide Metabolism

**Figure 4:**
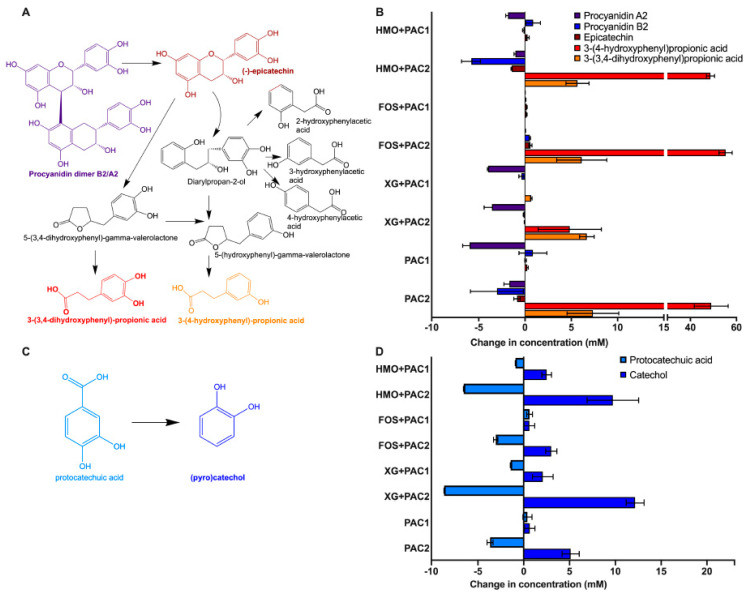
*Lactobacillus plantarum* degradation of phenolic compounds. Schematic diagram of potential degradation pathways of procyanidin A2 and B2 dimers (**A**) and the change in phenolic metabolite concentration following fermentation (**B**). Protocatechuic acid degradation into catechol (**C**) and the quantified change of their concentrations following fermentation (**D**). Averages from independent biological triplicates are shown, and bars represent standard deviation of the mean.

Procyanidins A2 and B2, flavan-3-ol dimers, and epicatechin (i.e., a monomeric flavan-3-ol) were quantified prior and subsequent to fermentation, along with several potential products of bacterial biotransformation (Figure 4A; Table 1). Uncharacterized precipitation was generally observed subsequent to the addition of PACs prior to fermentation. This appeared to be dependent on the oligosaccharide, and it is possible that the phenolic compound hydrophobicity is differentially impacted to form precipitants [40]. Therefore, the percent change from starting molecule concentrations is reported in Table 1.

All PAC2 fermentations resulted in 3-(3’,4’-dihydroxyphenyl)propionic acid and 3-(4’-hydroxyphenyl) propionic acid production (Figure 4B and Table 1). Bacteria are known to produce 3-(3’,4’-dihydroxyphenyl)propionic acid from procyanidin A2 and B2, epicatechins [5], and 3-(4’-hydroxyphenyl) propionic acid from procyanidin A2 degradation [41]. These metabolites were produced in a greater magnitude than the degradation of potential precursor products. This suggests that higher molecular weight PACs are degraded to end-products without a procyanidin B2 and A2 intermediate or other monomer/dimers.

Protocatechuic acid is a phenolic compound commonly found in plants and is decarboxylated to catechol (Figure 4C). Regardless of the oligosaccharide and PAC fraction, catechol concentrations increased during *L. plantarum* fermentations coinciding with a decrease in protocatechuic acid, with one exception (Figure 4D). Interestingly, both protocatechuic acid and catechol increased while *L. plantarum* utilized FOS in the presence of PAC1. In addition, increased epicatechin concentrations occurred during FOS fermentation with both PAC fractions with greater magnitudes than the other oligosaccharides (*p* < 0.05) and in the absence of *L. plantarum*, suggesting a degradation of PACs into epicatechin during FOS fermentation. It is not clear if this is linked to the PAC1 induction of FOS utilization (Figure 1B).

All PAC2 media contain high concentrations of p-coumaric acid prior to fermentation (~24–30 mM; Table 1). *L. plantarum* fermentations in the presence of PAC2 significantly decreased p-coumaric acid concentrations (88.59% ± 1.26%–99.50% ± 0.07%; Table 1). Conversely, p-coumaric acid increased spontaneously in PAC2 media in the absence of bacterial inoculation (Table S5).

### 3.5. PACs Generally Shift L. plantarum Transcriptomes in Concordance with Growth and Metabolic Phenotypes

**Figure 5:**
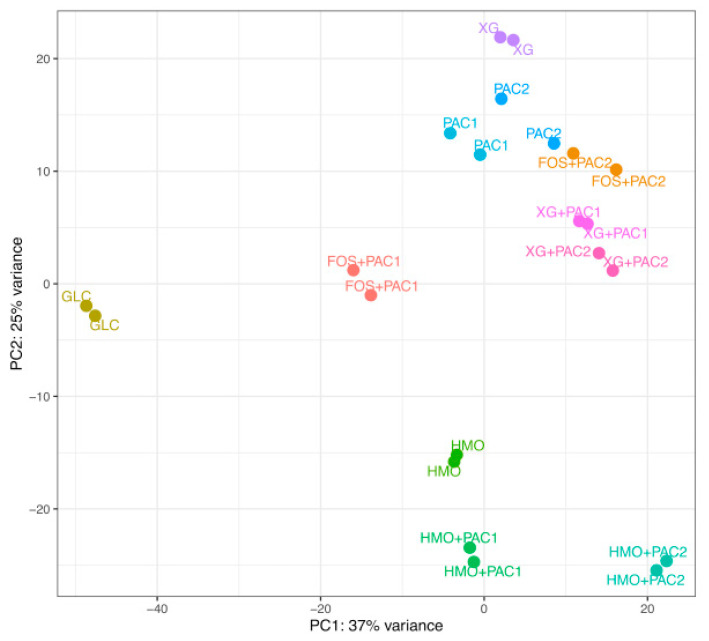
Principal component analysis of the *Lactobacillus plantarum* transcriptome response to cranberry substrates. Circles indicate distances between *L. plantarum* whole transcriptomes in response to glucose (GLC), cranberry proanthocyanidins (PACs) alone, fructooligosaccharides (FOS) with PACs, human milk oligosaccharides (HMO) with PACs, and xyloglucans (XG) with PACs.

Transcriptomes were generated from *L. plantarum* grown on dietary oligosaccharides in combination with PACs, yielding an average of 5.9 million RNA-seq paired-end reads. The alignment rate to the reference genome and coding regions, as well as total counts, are summarized in Table S6. Hierarchical clustering of the global transcriptomes based on Euclidean distances between samples is depicted in Figure S4. Whole transcriptomes were subjected to a principal component analysis (PCA) (Figure 5).

The HMO transcriptomes aggregate apart from the other culturing conditions, regardless of PACs. In contrast, most FOS and XG transcriptomes occupy similar and overlapping positions within the two-dimensional space of the PCA plot. *L. plantarum* utilizes FOS efficiently in the presence of PAC1 (Figure 1) to yield the most segregated transcriptome in the PCA, with the exception of glucose. PAC2 enables moderate growth on FOS to a similar magnitude as with PAC2 alone, which is mirrored in the proximity of their respective transcriptomes. This suggests that the limited PAC2-potentiated growth on FOS is potentially a function of utilizing PAC2 components to a greater extent than the oligosaccharide. *L. plantarum* ATCC BAA-793 is incapable of fermenting FOS alone and, thus, does not provide a transcriptome for comparison. Both PAC fractions enhance growth on xyloglucans similarly and induce near-superimposable transcriptional profiles almost indistinguishable from biological replicates of the same condition. Moreover, both PACs shift the XG transcriptomes along principal component 2 more significantly than the PAC influence on the HMO transcription. Additional transcriptome analyses, including impacts to the central fermentative pathway, carbohydrate metabolism, and glycosyl hydrolase expression, are reported in the Supplemental Information (Figures S5−S9).

### 3.6. Cranberry PACs Differentially Enrich Physiological Networks

A gene ontology (GO) enrichment analysis was performed to identify the biological processes, metabolic functions, and cellular components that respond to PACs in conjunction with oligosaccharides. The 10 highest expressed GO terms with normalized enrichment counts for each differential expression of log_2_ fold change are reported in Figures S10 and S11. While fermenting cranberry XG, both PAC extracts differentially regulated protein–sugar phosphotransferases and pyruvate-dependent sugar phosphotransferase systems (PTS) relative to glucose and XG alone (Figures S10B,C and S11A,B). Similarly, HMO+PAC2 induces PTS, carbohydrate binding, and ATP activity compared to glucose (Figure S10F). D-glucosamine PTS permease activity was enriched in both PACs compared to XG alone (Figure S11A,B), in HMO, HMO+PAC1, and FOS+PAC1 compared to glucose (Figure S10D,E,G). PACs in the absence of oligosaccharides increased the carbohydrate metabolic processes and predicted the methyl-beta-D-glucoside 6-phosphate glucohydrolase and 6-phospho-beta-glucosidase genes relative to glucose (Figure S10I,J). Interestingly, PAC2 increased the carbohydrate metabolic processes compared to XG alone (Figure S11B), which is potentially linked with the increase in acetic acid and formic acid production.

The GO analysis predicts that PAC2 increases Mo-molybdopterin cofactor biosynthesis in XG relative to glucose (Figure S10C). Relative to glucose, HMO, HMO+PAC1 (Figure S10D,E), and FOS+PAC2 (Figure S10H) exhibit a similar profile in inducing Mo-molybdopterin cofactor biosynthesis. In addition, Mo-molybdopterin cofactor biosynthesis was induced by both PAC fractions compared to HMO alone (Figure S11D,E). Molybdopterin is a cofactor for a diverse group of enzymes involved in redox reactions, including nitrate reductase [42].

During FOS fermentations, PAC1 increased the predicted acetyl-coenzyme A (CoA) carboxylase activity, whereas PAC2 induced a carbohydrate metabolic process compared to glucose (Figure S10G,H).

### 3.7. Polyphenol-Linked Genes Are Differentially Expressed during Synergistic Fermentations of Oligosaccharides with PACs

**Figure 6:**
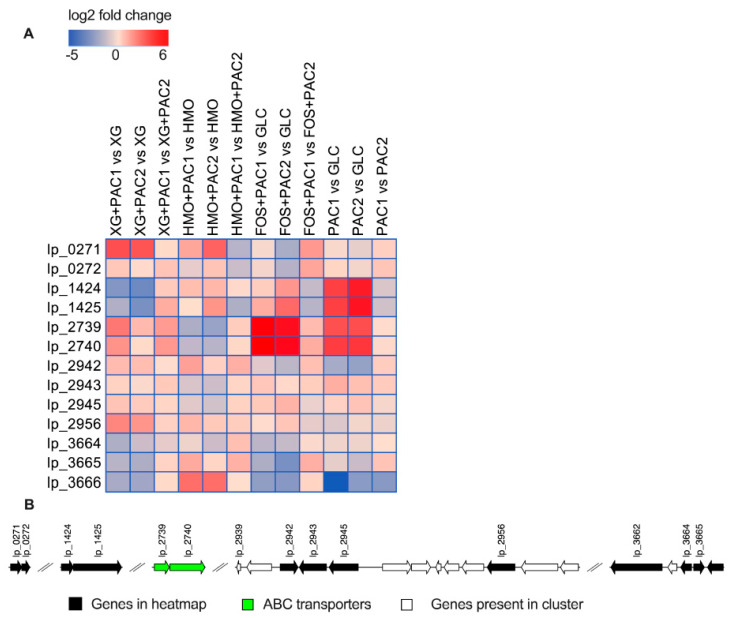
Relative gene expression of the predicted polyphenol metabolism genes within the global transcriptome. The log_2_ fold change gene expression from independent biological duplicates was performed from raw reads using the R package DESeq2 and is depicted in the heatmap (**A**). Potential genomic clusters involved in *L. plantarum* polyphenol utilization (**B**).

*L. plantarum* tannase and gallate decarboxylase enzymes respond to tannin degradation [43,44], and their differential expression is reported in Figure 6. Tannase (lp_2956) was upregulated in the presence of PACs during XG (log_2_ fold change = 2.0 and 1.7) and HMO (log_2_ fold change = 0.9 and 0.4) fermentation (*p* < 0.05). PAC1 upregulated a putative tannase transcriptional regulator (*tanR*; lp_2942) during XG (log_2_ fold change = 0.8) and HMO (log_2_ fold change = 1.4) fermentation relative to the corresponding oligosaccharides alone (*p* < 0.05).

*L. plantarum* uses gallate decarboxylase activity to degrade tannins potentially involving a putative decarboxylase (lp_2945) and an aromatic acid decarboxylase (lp_0272) [43]. Accordingly, PAC1 added to XG fermentations significantly increased lp_2945 expression relative to XG alone (log_2_ fold change = 0.6; *p* < 0.05), whereas it was significantly downregulated in HMO fermentation (log_2_ fold change = −0.7 and −1.0; *p* < 0.05). The gallate decarboxylase is co-transcribed with a transport protein (*gacP*; lp_2943) and *tanR* transcriptional regulator (lp_2942) genes [44]. The transport protein lp_2943 expression did not change during XG fermentation, although it was significantly downregulated in HMO fermentation (log_2_ fold change = −0.9 and −1.1; *p* < 0.05).

Nicotinamide adenine dinucleotide + hydrogen (NADH)-dependent flavin mononucleotide (FMN) reductase (lp_1424) and fumarate reductase/succinate dehydrogenase (lp_1425) were previously identified as hydroxycinnamic reductases and induced by p-coumaric acid [45]. While lp_1424 expression remained constant in the presence of PACs relative to HMO alone (*p* > 0.05), HMO+PAC2 prompted an increase in fumarate reductase (lp_1425) expression (log_2_ fold change = 1.7; *p* < 0.05), suggesting a functional link between polyphenol degradation and HMO metabolism (*p* < 0.05). These two genes were significantly downregulated by both PACs relative to XG alone (log_2_ fold change < −1.7; *p* < 0.05). There are additional reductases in the ATCC BAA-793 genome that may play a role in polyphenol transformation, including a nicotinamide adenine dinucleotide phosphate (NADPH)-dependent FMN-binding protein (lp_3490) that was upregulated by PAC2 fractions in both XG and HMO fermentation (log_2_ fold change = 1.9 and 1.5; *p* < 0.05; Figure S12). Similarly, two putative genes involved in fumarate reduction (lp_1112 and lp_1113) were upregulated during HMO+PAC2 metabolism compared to HMO alone (*p* < 0.05; Figure S12).

During FOS fermentation, a putative tannase (*tanB*; lp_2956) was upregulated by PAC2 (log_2_ fold change = 0.6, *p* < 0.05), and the *tanR* transcriptional regulator (lp_2942) was downregulated (log_2_ fold change = −1.4; *p* < 0.05). The PAC1 fraction alone significantly upregulated a transport protein (*gacP*; lp_2943; log_2_ fold change = 1.1; *p* < 0.05) while downregulating lp_2956 and lp_2942 (*p* < 0.05).

Putative transporters (lp_2739 and lp_2740) that potentially secrete pyrogallol after gallate degradation from tannins [44] were significantly upregulated by FOS with PACs (log_2_ fold change >4.0; *p* < 0.05). This is interesting, as these transporters were significantly downregulated by both fractions during HMO fermentation (log_2_ fold change < −1.5; *p* < 0.05). In XG fermentation, both PACs increase lp_2739, and PAC1 significantly increased lp_2740 expression (*p* < 0.05). The gallate decarboxylase lp_2945 was significantly induced by PACs during FOS fermentation (log_2_ fold change > 0.5; *p* < 0.05) and PAC2 alone (log_2_ fold change = 0.6; *p* < 0.05). The expression of the aromatic acid decarboxylase lp_0272 did not change significantly. A phenolic acid decarboxylase (*padA*; lp_3665) was downregulated by both fractions during FOS fermentation (*p* < 0.05).

The FMN reductase (lp_1424) and fumarate reductase (lp_1425) was significantly upregulated by FOS+PAC2 (log_2_ fold change > 1.5) and when PACs were added to the medium without carbohydrates (log2 fold change > 3.5). Similarly, the FMN-binding protein lp_3490 was upregulated by both PACs alone and PACs during FOS fermentation (*p* < 0.05; Figure S12), whereas the fumarate-related genes (lp_3491, lp_1112, and lp_1113) were significantly upregulated during FOS+PAC2 fermentations relative to glucose (*p* < 0.05; Figure S12).

FOS+PAC1 upregulated a 4-carboxymuconolactone decarboxylase that may be active in protocatechuate degradation (lp_2852; Figure S13A). This putative gene, along with an adjacent locus potentially related to quercetin dioxygenase (lp_2853) and an oxidoreductase (lp_2851), were downregulated by FOS+PAC1 relative to FOS+PAC2 (*p* < 0.05; Figure S13C). These three genes were upregulated by both PAC fractions during HMO fermentations compared to oligosaccharides alone (log_2_ fold change > 6; *p* < 0.05; Figure S12).

## 4. Discussion

In addition to directly impacting the host physiology, dietary polyphenols and oligosaccharides interact with gut microbiota with potential health outcomes. These molecules modulate specific microbial commensal populations to influence the emergent properties of the microbiome. Polyphenols are derived primarily from plants and, as such, compose whole foods or extracts and could impact the microbiome simultaneously. This includes cranberries, which contain an assortment of polyphenolic compounds and dietary fibers.

Previous studies investigated the microbial catabolism of proanthocyanidins and plant oligosaccharides separately using anaerobic bioreactors seeded with fecal extracts [46,47]. Moreover, prebiotic xylooligosaccharides and polyphenols extracted from several sources were studied in an in vitro human colon model to characterize fluxes to microbial properties [48]. Our previous study identified a potential relationship between polyphenols and cranberry oligosaccharides in bifidobacteria [16]. Therefore, we posited that cranberry components act synergistically as they are co-incorporated into whole foods and extracts. *L. plantarum* was selected to study as this organism is used as a probiotic in several applications, with a well-characterized function in maximizing the nutritional value of fermented food [10].

*L. plantarum* does not accumulate biomass efficiently in the absence of a fermentable carbohydrate. Interestingly, the PAC1 fraction isolated from cranberries enables *L. plantarum* growth in the absence of other sugars. PAC2 does so to a lesser extent, albeit close to the negative control medium that lacks carbohydrates. Despite a higher growth, it is notable that PAC1 has a lower carbohydrate content, both in soluble and glycoside forms (Table S1). PAC1 increased the growth efficiency during the fermentation of all the oligosaccharides tested. In contrast, PAC2 significantly increased the XG fermentation efficiency, which is interesting, as both the carbohydrate source and polyphenols were extracted from cranberries.

In contrast to PAC1, PAC2 promoted a minor growth enhancement during FOS fermentation, similar to what was observed on PAC2 alone (Figure 2E). Moreover, PAC2 induced higher concentrations of lactic acid in cultures while utilizing FOS, despite PAC1 eliciting a much greater *L. plantarum* biomass. The XG and HMO fermentations yielded higher end-product secretions relative to FOS utilization (Figure 2). We hypothesize that *L. plantarum* FOS utilization does not proceed through fermentation but, rather, anaerobic respiration. The high biomass observed during FOS+PAC1 utilization in the absence of the predicted fermentation end-products supports this. As FOS+PAC2 resulted in a low biomass, and with the similar low end-product concentrations observed with PAC1, it is possible that there is minor fermentation of the trace carbohydrates introduced by the PAC fractions.

Genes that may play a role in anaerobic respiration have been previously identified in *L. plantarum*, including those that enable the use of nitrate and nitrite as electron acceptors [49,50]. Moreover, it has been reported that *L. plantarum* utilizes inaccessible carbon sources such as mannitol when anaerobic electron acceptors are added to the medium [51]. Although anaerobic respiration has been previously documented in lactobacilli, the observations presented herein prompted the hypothesis that anaerobic respiration proceeds during FOS utilization in the presence of PACs with possible terminal electron acceptors, including DMSO. As PAC1 induced the phenotype, and not PAC2, the solvent DMSO is not solely responsible for this phenomenon. The formate produced from the fermentation of residual carbohydrates could donate electrons to DMSO, as previously observed in other systems [52]. More likely, and typically expected during respiration, NADH is the electron donor, and an NADH dehydrogenase (lp_0313, *ndh1*) was upregulated during FOS+PAC1 utilization (log_2_ fold change = 0.96; *p* < 0.05). The visualization of differential gene expressions on a volcano plot indicated that a glycerol kinase (*glpK*; lp_0370) and a glycerol-3-phosphate dehydrogenase (*glpD*; lp_0371) were upregulated during FOS+PAC1 utilization (Figure S13). This is significant, as anaerobic respiration may couple glycerol-3-phosphate dehydrogenase with fumarate reductase or formate dehydrogenase [53]. Furthermore, genes related to redox reactions, including molybdopterin biosynthesis, fumarate reductase, and nitrate reductase, were differentially regulated in the presence of cranberry PACs and may participate in respiration.

In previous in vitro modeled microbiome research, procyanidin dimers were degraded to phenolic metabolites with profiles similar to the products observed in this study [41,54,55]. In addition, 3-(4’-hydroxyphenyl) propionic acid, a metabolite of p-coumaric acid [45], was detected in most PAC1 fermentations, with the exception of XG+PAC1. This is consistent with the increase in p-coumaric acid (instead of degradation) during XG+PAC1 fermentation. This suggests that *L. plantarum* metabolizes or transforms p-coumaric acid, potentially through previously characterized decarboxylase activity. Interestingly, p-coumaric acid increased during XG+PAC1 fermentations, which may be the result of anthocyanin degradation [56].

Hydroxycinnamic acid reductase (lp_1425) is upregulated in most PAC2 fermentations, with the exception of XG+PAC2. Moreover, a putative protocatechuic acid decarboxylase (lp_2852) is upregulated, except during XG+PAC1 fermentation. This indicates that the cranberry-derived XG modifies the transcriptional response to cranberry p-coumaric and protocatechuic acids. Understanding the significance of this is of potential value in the context of a whole food approach to nutrition.

The underlying mechanism by which cranberry polyphenols act synergistically with dietary oligosaccharides is not currently understood. A potential contributing factor is that polyphenols modulate the transport of exogenous carbohydrates. A previous report speculated that this may occur during *L. plantarum* RM71 galactose fermentation in the presence of catechin to result in greater substrate utilization and lactic acid production [57]. This is consistent to what was observed in the present study, in that PACs increased the transcription of genes annotated as ABC transporters, permeases, and PTS-coupled sugar transport. Increased transport potential may lead to a greater intracellular availability of fermentable substrates.

Both PAC fractions are enriched in proanthocyanidins, and PAC2 contains 15-fold higher total anthocyanins than PAC1. All occurring anthocyanins are O-glycosylated with carbohydrate moieties [58]. Therefore, *L. plantarum* may hydrolyze these glycan linkages to liberate carbohydrates for fermentation. This, however, is not likely the primary driver of the synergistic influence of cranberry PACs with oligosaccharides. Accordingly, the PAC1 fraction exhibits a much more pronounced growth enhancement, despite less residual carbohydrates. PAC2 approaches the PAC1 synergistic impact only in facilitating cranberry xyloglucan fermentation.

*L. plantarum* utilizes dietary oligosaccharides more efficiently in the presence of cranberry polyphenols. There are clear impacts on the cellular physiology and links to global transcription. It is apparent that this probiotic microbe hydrolyzes cranberry polyphenols as well. It is unclear, however, if and how this synergism may impact the human gut microbiome and, ultimately, the host. Characterizing the underlying physiological principles in mechanistic detail will provide greater clarity. This may inform next-generation synergistic synbiotic approaches that incorporate adjunct substrates such as cranberry polyphenols.

## Figures and Tables

**Table 1:** Cranberry phenolic metabolites produced during *Lactobacillus plantarum* oligosaccharide fermentation ^a.^

	**PACs in the Absence of Fermentable Carbohydrates**							
	**PAC1 Pre**	**PAC1 Post**	**Delta**	**% Change**	**PAC2 Pre**	**PAC2 Post**	**Delta**	**% Change**
Procyanidin A2	8.60	2.59 ± 0.66	−6.01 ± 0.66	−69.86 ± 7.68	6.01	4.31 ± 0.59	−1.70 ± 0.59	−28.31 ± 9.86
Procyanidin B2	0.02	0.88 ± 1.51	0.86 ± 1.51	NA	5.99	2.95 ± 2.86	−3.04 ± 2.86	−50.73 ± 47.85
Epicatechin	0.29	0.32 ± 0.04	0.03 ± 0.04	8.73 ± 13.79b	1.19	0.81 ± 0.15	−0.38 ± 0.15	−31.65 ± 12.75b
3-(3,4-dihydroxyphenyl)propionic acid	0.00	0.00 ± 0.00	NA	NA	0.09	7.89 ± 2.97	7.80 ± 2.97	8558 ± 3254
3-(4-hydroxyphenyl)propionic acid	0.00	0.24 ± 0.10	0.24 ±0.10	NA	0.20	48.38 ± 7.25	48.18 ± 7.25	24,054 ± 3618a
3,4-dihydroxyphenylacetic acid	0.02	0.02 ± 0.04	0.00 ± 0.00	−1.82 ± 170.05	0.02	0.06 ± 0.02	0.04 ± 0.02	144.30 ± 88.06
4-hydroxyphenylacetic acid	0.50	0.37 ± 0.18	−0.13 ± 0.18	−25.09 ± 35.96	0.46	0.47 ± 0.15	0.01 ± 0.15	0.71 ± 33.42
Protocatechuic acid	1.48	1.95 ± 0.59	0.47 ± 0.59	31.98 ± 35.91a *	7.31	2.97 ± 0.38	−4.35 ± 0.59	−59.45 ± 5.21 *
Catechol	0.00	0.65 ± 0.56	0.65 ± 0.56	NA	0.00	5.04 ± 0.92	5.04 ± 0.92	NA
Syringic acid	0.10	0.09 ± 0.02	−0.01 ± 0.02	−9.50 ± 20.14 *	0.02	0.21 ± 0.08	0.19 ± 0.08	1049 ± 451b *
p-coumaric acid	0.02	0.00 ±0.00	−0.01 ± 0.00	−79.77 ± 17.52b	29.51	0.15 ± 0.02	−29.36 ± 0.02	−99.50 ± 0.06
	**Xyloglucans**							
	**PAC1 Pre**	**PAC1 Post**	**Delta**	**% Change**	**PAC2 Pre**	**PAC2 Post**	**Delta**	**% Change**
Procyanidin A2	4.38	0.36 ± 0.06	−4.02 ±0.06	−91.71 ± 1.45	6.83	3.25 ± 0.85	−3.58 ± 0.85	−52.40 ± 12.51
Procyanidin B2	0.36	0.08 ± 0.07	−0.28 ±0.07	−78.24 ± 18.86	0.41	0.25 ± 0.05	−0.16 ± 0.05	−38.83 ± 11.24
Epicatechin	0.07	0.08 ± 0.00	0.01 ± 0.00	13.89 ± 4.81b	0.23	0.18 ± 0.04	−0.05 ± 0.04	−21.37 ± 12.75b
3-(3,4-dihydroxyphenyl)propionic acid	0.00	0.18 ± 0.10	0.18 ± 0.10	NA	0.14	6.98 ± 0.80	6.84 ± 0.80	4815 ± 566
3-(4-hydroxyphenyl)propionic acid	0.00	0.00 ± 0.00	0.00 ± 0.00	NA	1.65	2.65 ± 0.38	1.00 ± 0.38	60.38 ± 23.19c
3,4-dihydroxyphenylacetic acid	0.00	0.00 ± 0.00	0.00 ± 0.00	NA	0.00	0.00 ± 0.00	0.00 ± 0.00	NA
4-hydroxyphenylacetic acid	0.00	0.06 ± 0.05	0.06 ± 0.05	NA	0.00	0.06 ± 0.05	0.06 ± 0.05	NA
Protocatechuic acid	1.67	0.26 ± 0.03	−1.41 ± 0.03	−84.60 ± 1.93b	8.72	0.18 ± 0.01	−8.54 ± 0.01	−97.74 ± 0.08
Catechol	0.27	2.42 ± 1.16	2.15 ± 1.16	800 ± 433	0.00	11.34 ± 1.01	11.34 ± 1.01	NA
Syringic acid	0.17	0.31 ± 0.10	0.14 ± 0.10	79.63 ± 58.88	0.33	0.29 ± 0.09	−0.04 ± 0.09	−11.43 ± 27.26c
p-coumaric acid	0.04	1.60 ± 0.08	1.56 ± 0.08	3887 ± 197a *	23.82	2.72 ± 0.30	−21.12 ± 0.30	−88.59 ± 1.26 *
	**Fructooligosaccharides**							
	**PAC1 Pre**	**PAC1 Post**	**Delta**	**% Change**	**PAC2 Pre**	**PAC2 Post**	**Delta**	**% Change**
Procyanidin A2	0.00	0.04 ± 0.04	0.04 ± 0.04	NA	0.04	0.04 ± 0.04	0.00 ± 0.04	−2.03 ± 100.69
Procyanidin B2	0.03	0.09 ± 0.03	0.06 ± 0.03	207 ± 113	0.07	0.59 ± 0.07	0.52 ± 0.07	719.11 ± 94.31
Epicatechin	0.05	0.13 ± 0.02	0.08 ± 0.02	163.37 ± 30.82a	0.17	0.42 ± 0.07	0.26 ± 0.07	155.60 ± 46.27a
3-(3,4-dihydroxyphenyl)propionic acid	0.04	0.00 ± 0.00	−0.04 ± 0.00	−100 ±0	0.00	6.51 ± 2.88	6.51 ± 2.88	NA
3-(4-hydroxyphenyl)propionic acid	0.00	0.16 ± 0.06	0.16 ± 0.06	NA	0.20	54.55 ± 2.79	54.35 ± 2.79	26,959 ± 1386a
3,4-dihydroxyphenylacetic acid	0.03	0.02 ± 0.03	−0.02 ± 0.03	−56.42 ± 75.48	0.04	0.09 ± 0.07	0.05 ± 0.07	115.68 ± 155.38
4-hydroxyphenylacetic acid	0.36	0.36 ±0.13	−0.01 ± 0.13	−1.50 ± 34.50	0.40	0.54 ± 0.10	0.13 ± 0.10	31.60 ± 25.11
Protocatechuic acid	1.39	2.15 ± 0.38	0.75 ± 0.38	54.16 ± 27.16a *	6.26	2.64 ± 0.28	−3.62 ± 0.28	−57.79 ± 4.46 *
Catechol	0.00	0.61 ± 0.56	0.61 ± 0.56	NA	0.00	2.95 ± 0.61	2.95 ± 0.61	NA
Syringic acid	0.08	0.07 ± 0.05	−0.01 ± 0.05	−8.14 ± 56.34 *	0.01	0.18 ± 0.03	0.18 ± 0.03	3251 ± 553a *
p-coumaric acid	0.02	0.00 ± 0.00	−0.02 ± 0.00	−98.76 ± 0.07b	30.29	0.37 ± 0.02	−29.92 ± 0.02	−98.76 ± 94.31
	**Human Milk Oligosaccharides**							
	**PAC1 Pre**	**PAC1 Post**	**Delta**	**% Change**	**PAC2 Pre**	**PAC2 Post**	**Delta**	**% Change**
Procyanidin A2	5.90	4.05 ± 0.24	−1.85 ± 0.24	−31.34 ± 4.14	6.79	5.74 ± 0.14	−1.04 ± 0.14	−15.39 ± 2.13
Procyanidin B2	0.01	0.90 ± 0.79	0.88 ± 0.79	6306 ± 5641	6.99	1.19 ± 0.99	−5.80 ± 0.99	−83.04 ± 14.10
Epicatechin	0.28	0.21 ± 0.05	−0.07 ± 0.05	−25.45 ± 17.03b	1.13	0.50 ± 0.03	−0.63 ± 0.03	−55.69 ± 3.02b
3-(3,4-dihydroxyphenyl)propionic acid	0.00	0.00 ± 0.00	0.00 ± 0.00	NA	0.00	6.03 ± 1.32	6.03 ± 1.32	NA
3-(4-hydroxyphenyl)propionic acid	0.00	0.27 ± 0.16	0.27 ± 0.16	NA	0.37	48.25 ± 1.75	47.87 ± 1.75	12,846 ± 470b
3,4-dihydroxyphenylacetic acid	0.03	0.04 ± 0.01	0.01 ± 0.01	45.59 ± 34.87	0.08	0.14 ± 0.02	0.06 ±0.02	78.96 ± 27.39
4-hydroxyphenylacetic acid	1.67	2.50 ± 0.38	0.83 ± 0.38	49.81 ± 23.05	1.25	1.91 ± 0.32	0.66 ± 0.32	52.46 ± 25.40
Protocatechuic acid	1.23	0.17 ± 0.02	−1.07 ± 0.02	−86.46 ± 1.50b	7.91	0.27 ± 0.03	−7.65 ± 0.03	−96.59 ± 0.44
Catechol	0.00	2.46 ± 0.52	2.46 ± 0.52	NA	0.00	9.59 ± 2.77	9.59 ± 2.77	NA
Syringic acid	0.04	0.06 ± 0.02	0.02 ± 0.02	47.31 ± 48.26	0.03	0.25 ± 0.04	0.21 ± 0.04	639 ± 113bc
p-coumaric acid	0.00	0.00 ± 0.00	0.00 ± 0.00	−73.49 ± 45.92b	24.97	0.12 ± 0.02	−24.85 ± 0.02	−99.50 ± 0.07

^a^ Pre refers to the medium prior to *L. plantarum* inoculation, and post refers the supernatants subsequent to growth. The letters indicate significant differences observed in post-fermentation (i.e., percent change) using one-way ANOVA with Tukey’s test (*p* < 0.05) within the same proanthocyanidin (PAC) fraction. An asterisk indicates the significant difference between the PAC fractions within the same oligosaccharide.

## Data Availability

Raw reads are deposited in the NCBI Gene Expression Omnibus database (https://www.ncbi.nlm.nih.gov/geo/ (accessed on 19 March 2021)) under accession number GSE160565.

## Supplementary Materials

The following are available online at https://www.mdpi.com/2076-2607/9/3/656/s1: Figure S1: *Lactobacillus plantarum* growth while utilizing glucose in the presence of cranberry PACs. Figure S2: Bacterial growth while utilizing oligosaccharides with cranberry PACs. Figure S3: *Lactobacillus plantarum* secreted fermentative end-products while utilizing cranberry PACs alone. Figure S4: The *Lactobacillus plantarum* transcriptome response to carbohydrates and cranberry PACs. Figure S5: Galactose metabolism responding to HMO and PACs. Figure S6: Fructose metabolism responding to FOS in the presence of PAC fermentation. Figure S7: *Lactobacillus plantarum* carbon metabolism genes responding to the oligosaccharide and PACs. Figure S8: Relative gene expression of glycoside hydrolases within the global transcriptome. Figure S9: Gene expression within an arabinose and rhamnose utilization cluster in *Lactobacillus plantarum*. Figure S10: Top 10 Gene Ontology enrichment analyses of pairwise comparisons. Figure S11: Top 10 Gene Ontology enrichment analyses of pairwise comparisons. Figure S12: Relative gene expression of predicted fumarate reductases within the global transcriptome. Figure S13: Volcano plots of differential gene expression profiles of pairwise comparisons. Table S1: Composition of cranberry PAC extracts (Ocean Spray, personal communication). Table S2: MS/MS conditions for phenolic metabolite detection. Table S3: Growth kinetics of *Lactobacillus plantarum* ATCC BAA-793 with PACs calculated with Growthcurver. Table S4: Growth kinetics of other lactobacilli strains grown on carbohydrates with PACs calculated with Growthcurver. Table S5: Spontaneous degradation of phenolic compounds in the absence of *Lactobacillus plantarum*. Table S6: General RNA-seq transcriptome features.
