# Supplementary material for: Cranberry Proanthocyanidins and Dietary Oligosaccharides Synergistically Modulate Lactobacillus plantarum Physiology
Source: Microorganisms. 2021 Mar 22;9(3):656. doi: 10.3390/microorganisms9030656 (PMC8004764; doi:10.3390/microorganisms9030656)
Supplement: Supplementary file 1 [file microorganisms-09-00656-s001.zip › microorganisms-1129408.pdf]

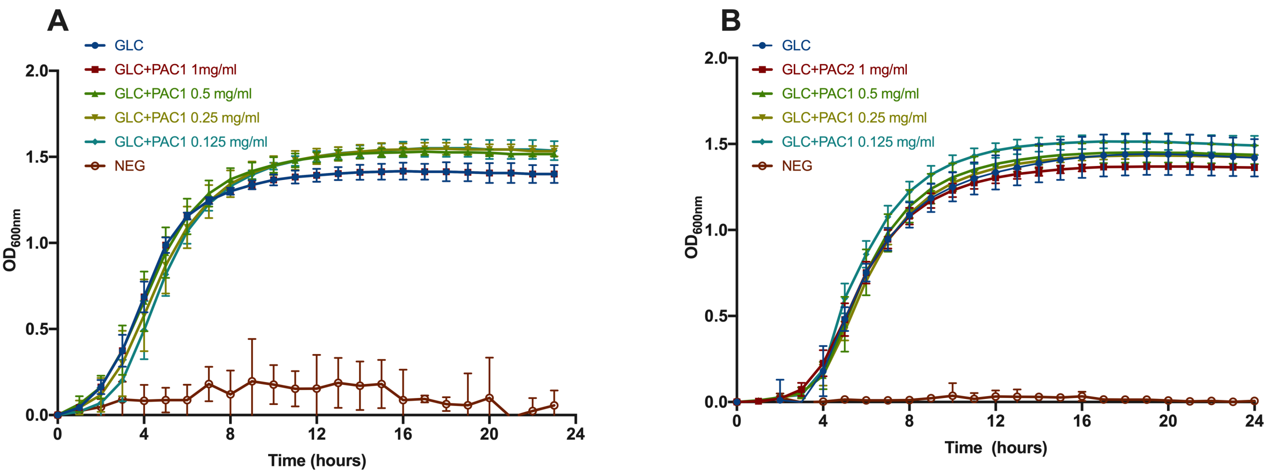

Fig. S1 *Lactobacillus plantarum* growth while utilizing glucose in the presence of cranberry PACs. Growth curves of *Lactobacillus plantarum* ATCC BAA-793 on modified MRS containing 0.125 - 1.0 mg/mL PAC1 (A) and PAC2 (B) fractions with 1% (w/v) glucose. The curves are drawn from average of three independent replicates.

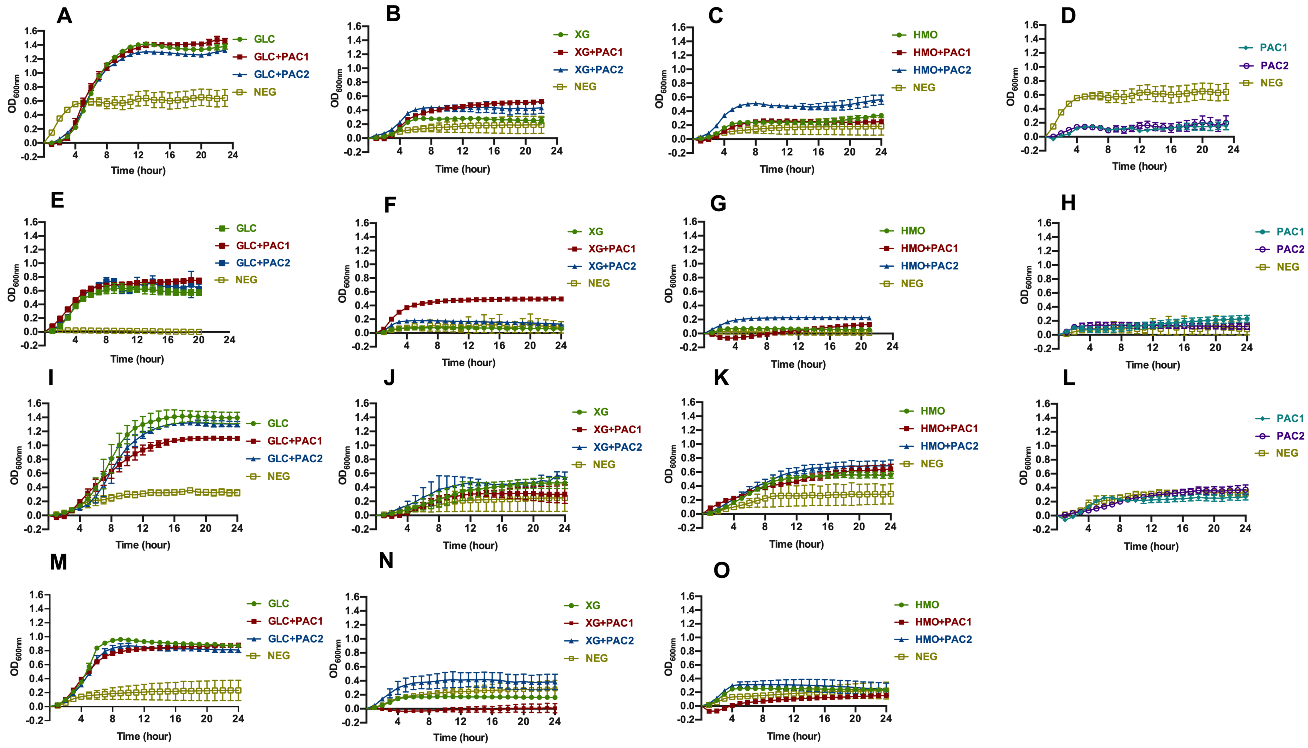

Fig. S2 Bacterial growth while utilizing oligosaccharides with cranberry PACs. Growth curves of *Lactobacillus plantarum* ATCC 14917(A-D), *Lactobacillus johnsonii* (E-H), *Lactobacillus pentosus* (I-L), and *Lactobacillus reuteri* (M-O) on modified MRS containing 1000 mg/ml PAC extracts and 1 % (w/v) glucose (A, E, I, M), xyloglucans (B, F, J, N), human milk oligosaccharides (C, G, K, O), PACs alone (D, H, L). The curves are drawn from average of three independent replicates.

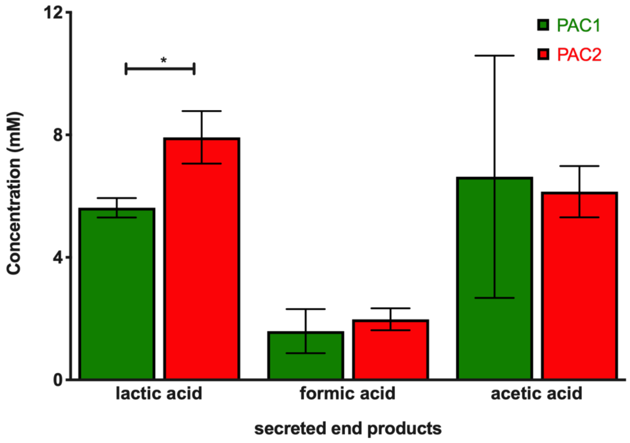

Fig S3 *Lactobacillus plantarum* secreted fermentative end-products while utilizing cranberry PACs alone. Absolute concentrations of lactic acid, acetic acid, and formic acid following fermentation PACs only. Averages from independent biological triplicates are depicted and bars represent standard deviations of the mean. The values for organic acid production are expressed in millimolar absolute concentration. Asterisks represent significant difference evaluated by two-way ANOVA and Tukey's multiple comparison test ($p<0.05$)

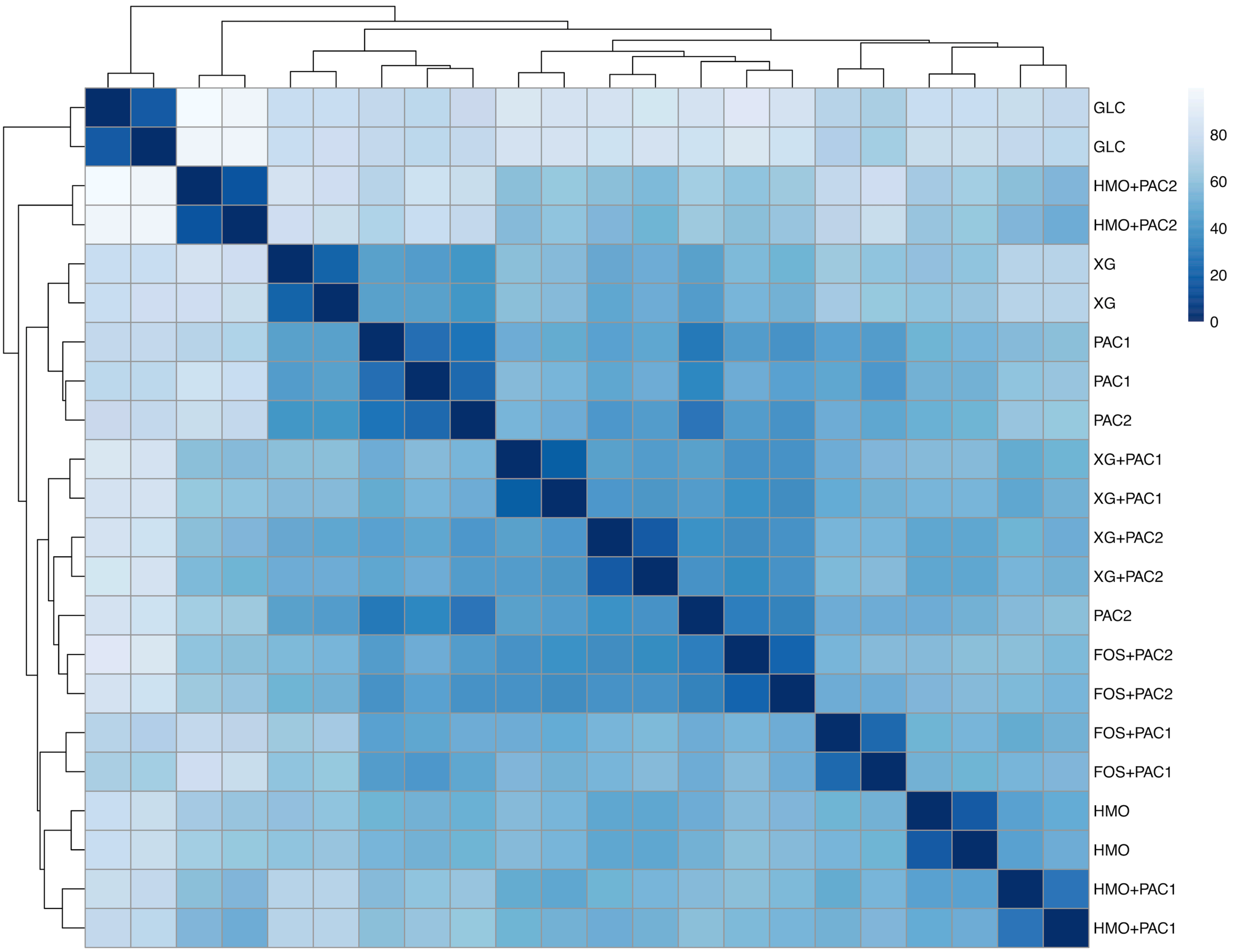

Fig. S4 The *Lactobacillus plantarum* transcriptome response to carbohydrates and cranberry PACs. The heatmap depicts the *L. plantarum* whole transcriptomes in response to glucose (GLC), cranberry proanthocyanidins (PACs) alone, fructooligosaccharides (FOS) with PACs, human milk oligosaccharides (HMO) with PACs and xyloglucans (XG) with PACs. Euclidian distances are calculated of the regularized log transformation of counts with biological duplicates. The blue color intensity indicates the degree of similarity, from white (dissimilar) to dark blue (most similar). Likewise, the dendograms depicts distances with larger branch lengths indicating greater distances.

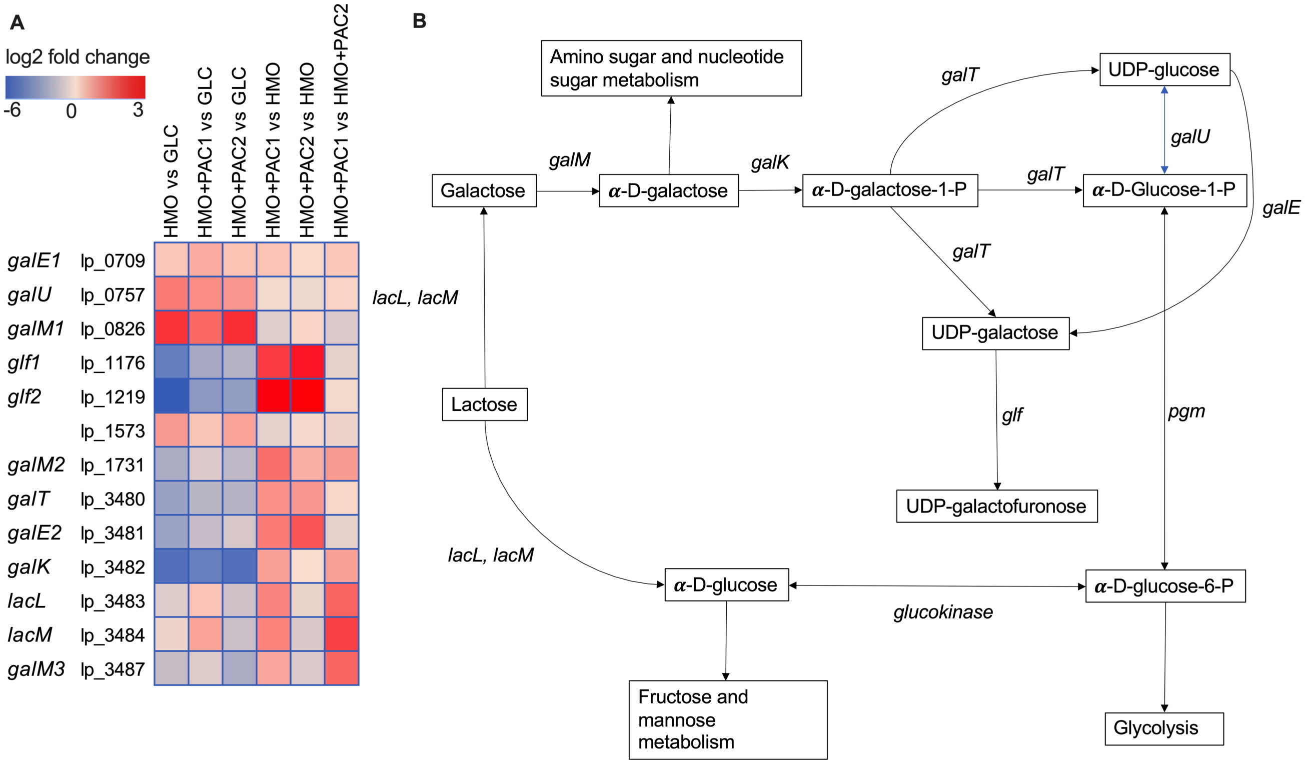

Fig. S5 Galactose metabolism responding to HMO and PACs. The differential expressions of genes in galactose metabolism (Leloir pathway) in *Lactobacillus plantarum* as $\log_2$ fold change are shown in the heatmap (A). Red denotes upregulation with blue corresponding to downregulation. The galactose metabolism pathway (B) is drawn based on KEGG pathways maps (https://www.genome.jp/kegg-bin/show).

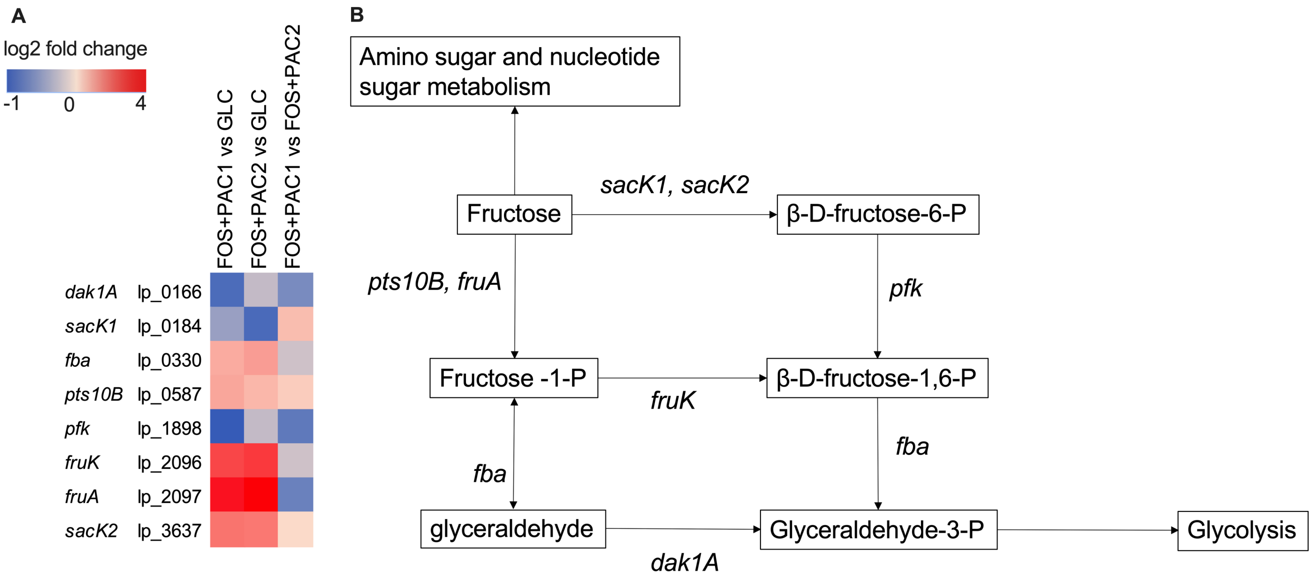

Fig S6 Fructose metabolism responds to FOS in the presence of PACs fermentation. The differential expressions of fructose-related genes as $\log_2$ fold change are shown in the heatmap (A). Red denotes upregulation with blue corresponding to downregulation. The fructose metabolism pathway (B) is drawn based on KEGG pathways maps.

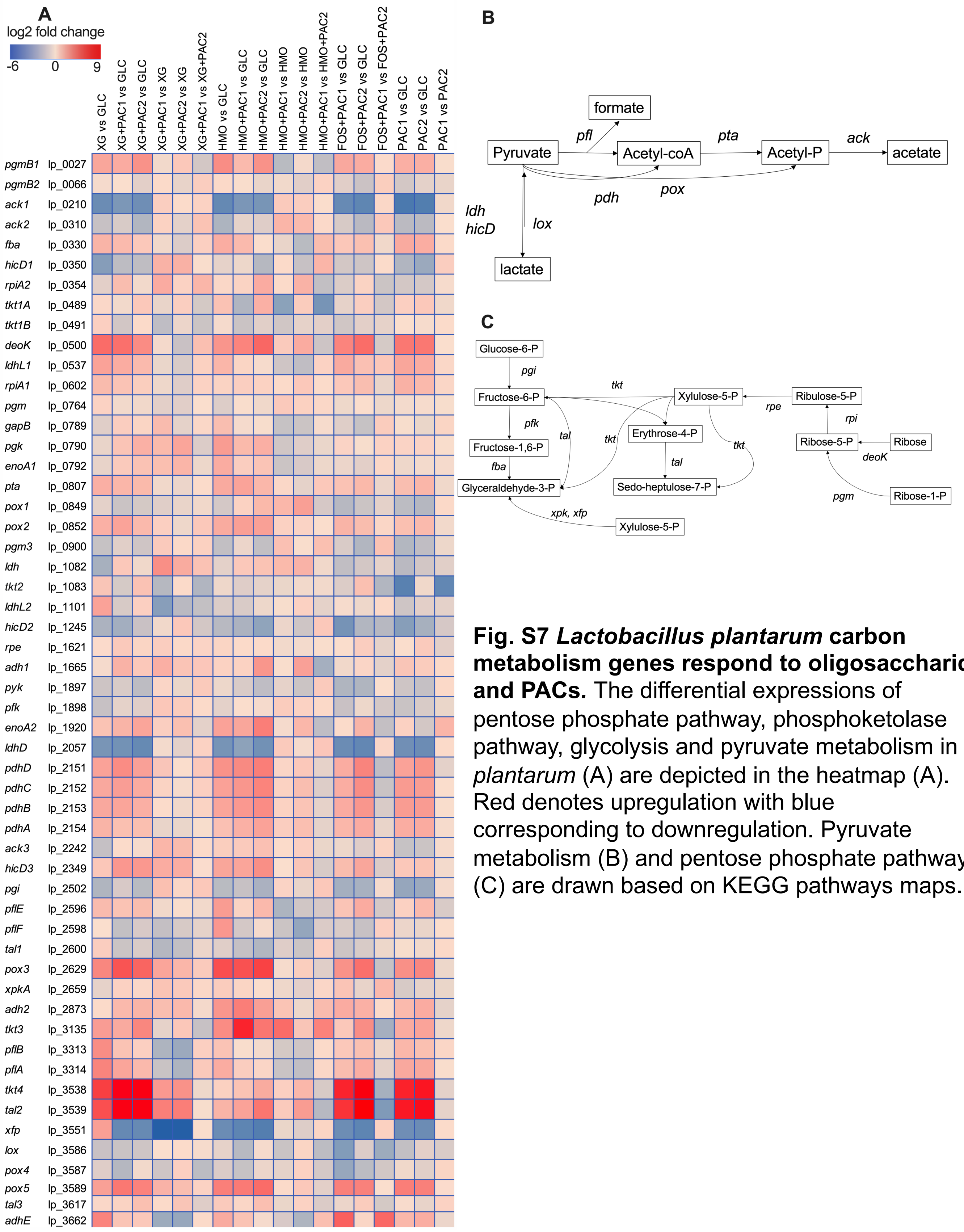

Fig. S7 *Lactobacillus plantarum* carbon metabolism genes respond to oligosaccharide and PACs*.* The differential expressions of pentose phosphate pathway, phosphoketolase pathway, glycolysis and pyruvate metabolism in *L. plantarum* (A) are depicted in the heatmap (A). Red denotes upregulation with blue corresponding to downregulation. Pyruvate metabolism (B) and pentose phosphate pathway (C) are drawn based on KEGG pathways maps.

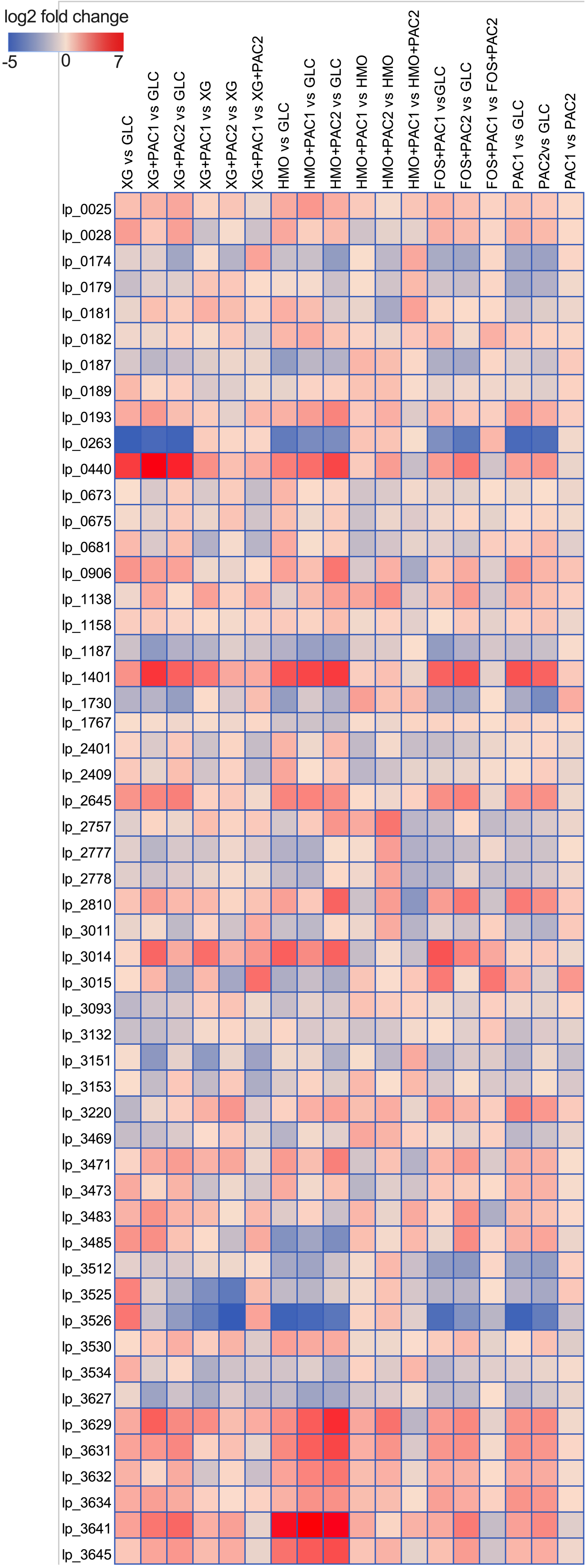

Fig S8 Relative gene expression of glycoside hydrolases within the global transcriptome. The $\log_2$ fold change gene expression of glycoside hydrolases (GH) obtained from CAZY database from independent biological duplicates was performed from raw reads using the R package DESeq2.

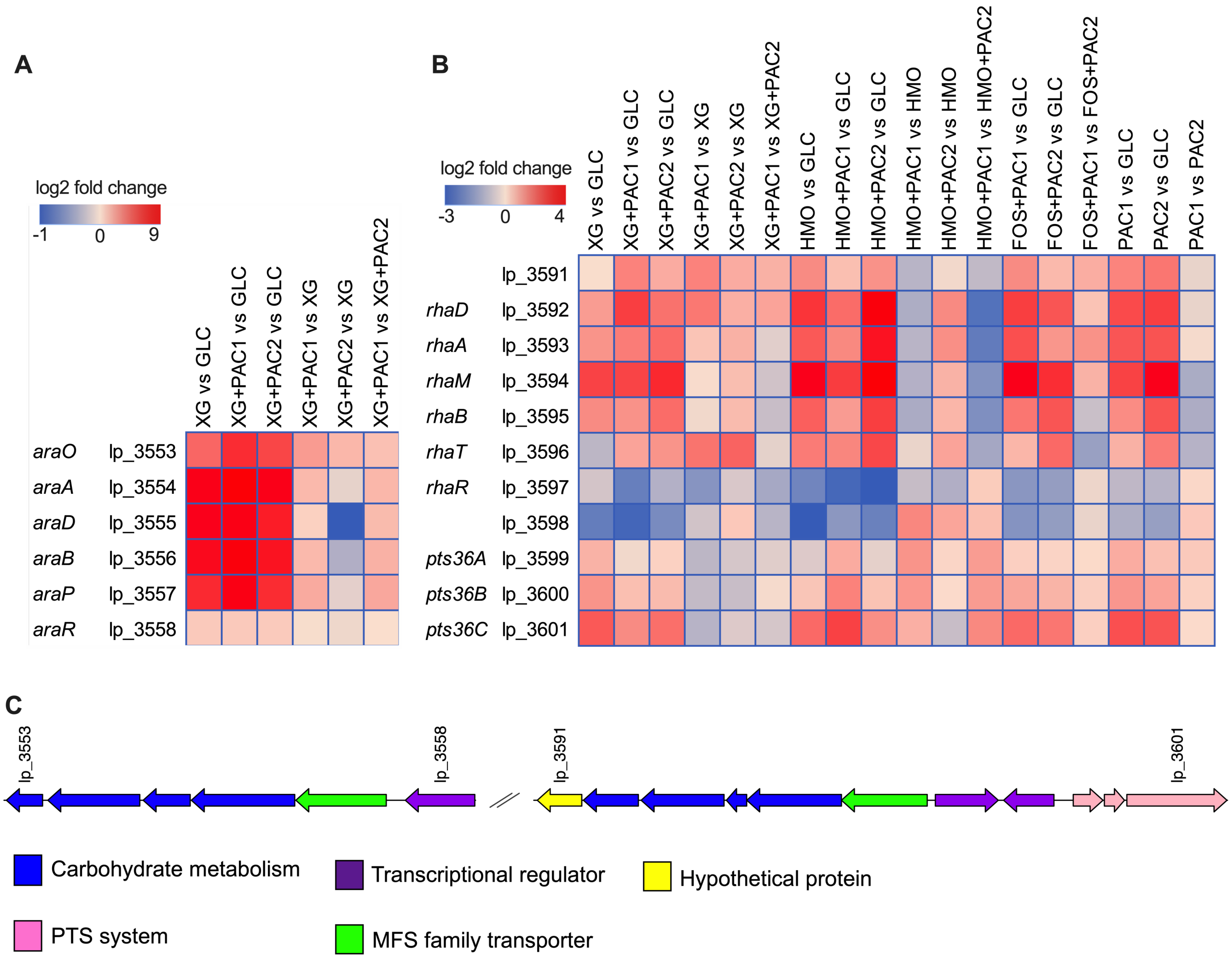

Fig S9 Gene expression within an arabinose and rhamnose utilization cluster in *Lactobacillus plantarum.* Log$_2$ fold change of arabinose utilization cluster genes in xyloglucan fermentations (A) and rhamnose cluster in all fermentations (B). The genomic architecture of the *L. plantarum* arabinose and rhamnose utilization clusters (C).

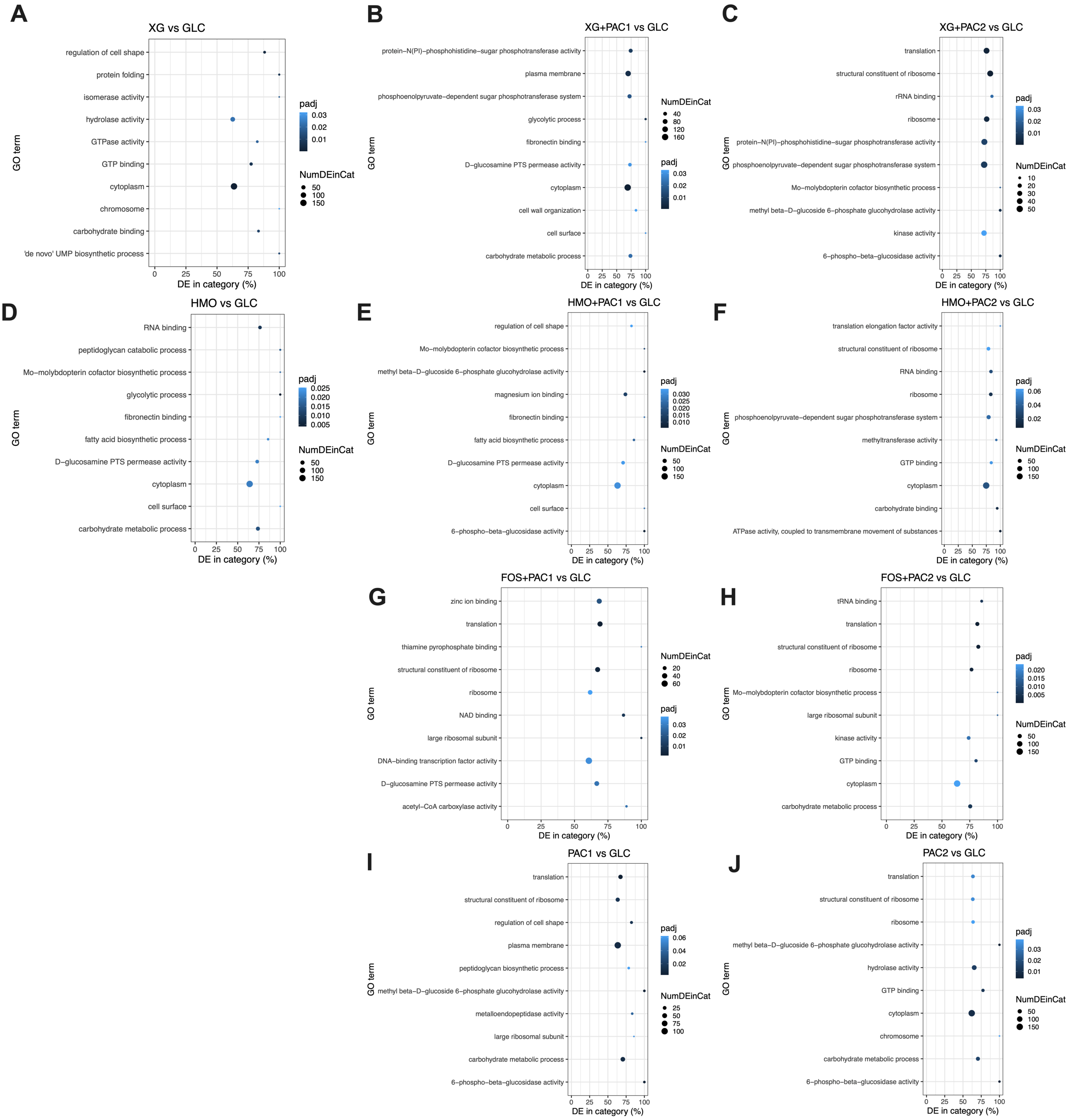

Fig S10 Top 10 Gene Ontology enrichment analysis of pairwise comparisons. The differential expressions of Gene Ontology (GO) terms are shown as percent number of differentially expressed gene counts in each GO category for *Lactobacillus plantarum* growing on xyloglucans (XG) with PAC1 (A), PAC2 (B) versus XG, XG+PAC2 vs XG+PAC1 (C) and human milk oligosaccharides (HMO) with PAC1 (D), PAC2 (E) versus HMO, and HMO+PAC2 versus HMO+PAC1 (F), PAC1 versus glucose(GLC) (G), PAC2 versus GLC (H), and PAC2 versus PAC1(I). p values are calculated by the R package GOSeq. The number in each bubble indicates the count of differentially expressed genes in the GO category.

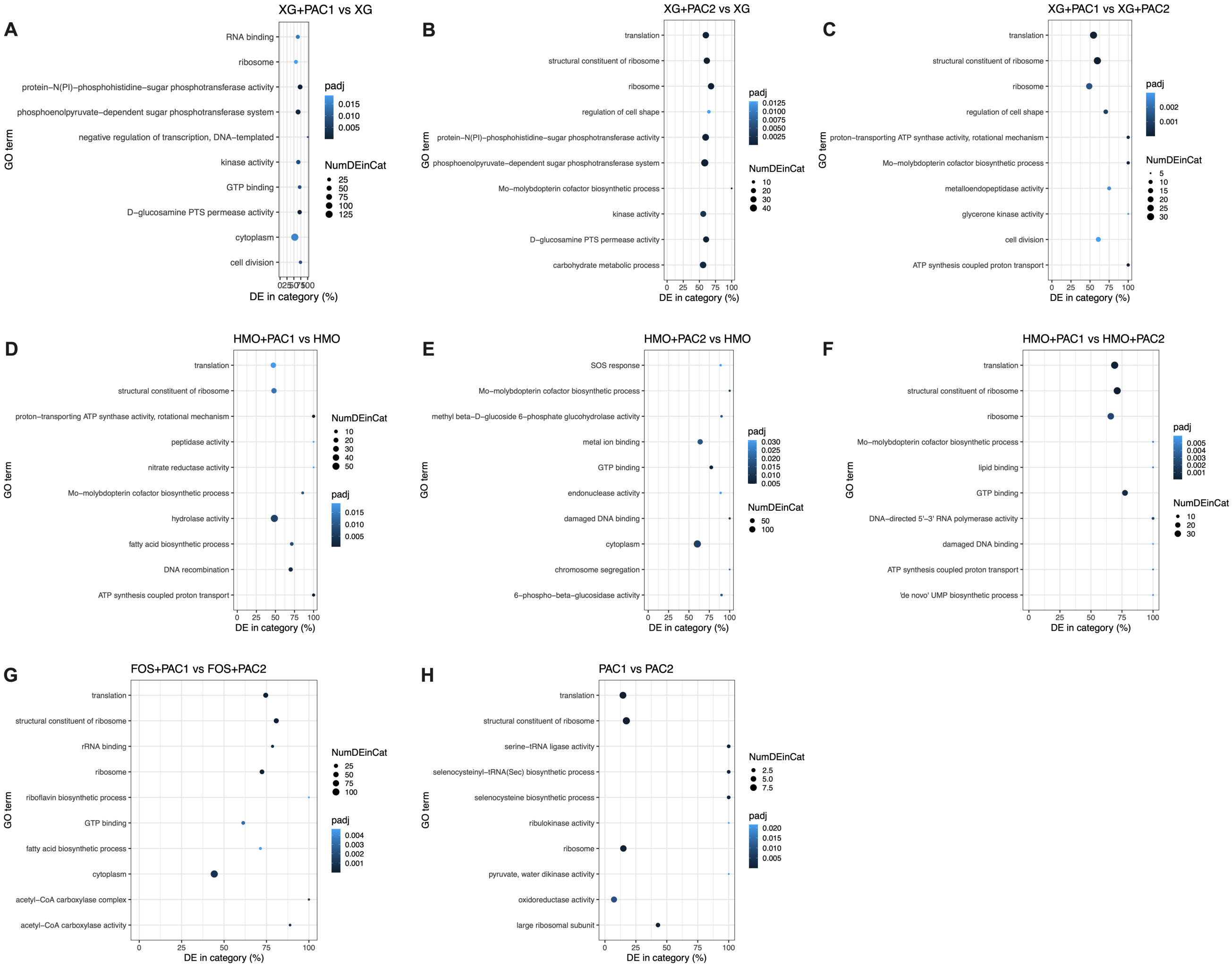

Fig S11 Top 10 Gene Ontology enrichment analysis of pairwise comparisons. The differential expressions of GO terms are shown percent number of differentially expressed gene counts in each GO category for *Lactobacillus plantarum* growing in xyloglucan (XG) with PAC1 (A), PAC2 (B) versus XG, XG+PAC1 vs XG+PAC2 (C) and human milk oligosaccharides (HMO) with PAC1 (D), PAC2 (E) versus HMO, and HMO+PAC1 versus HMO+PAC2 (F), FOS+PAC1 vs FOS+PAC2 (G), PAC1 vs PAC2(H). Adjusted p values are calculated by GOSeq. The number in each bubble indicate the count of differentially expressed genes in the GO category.

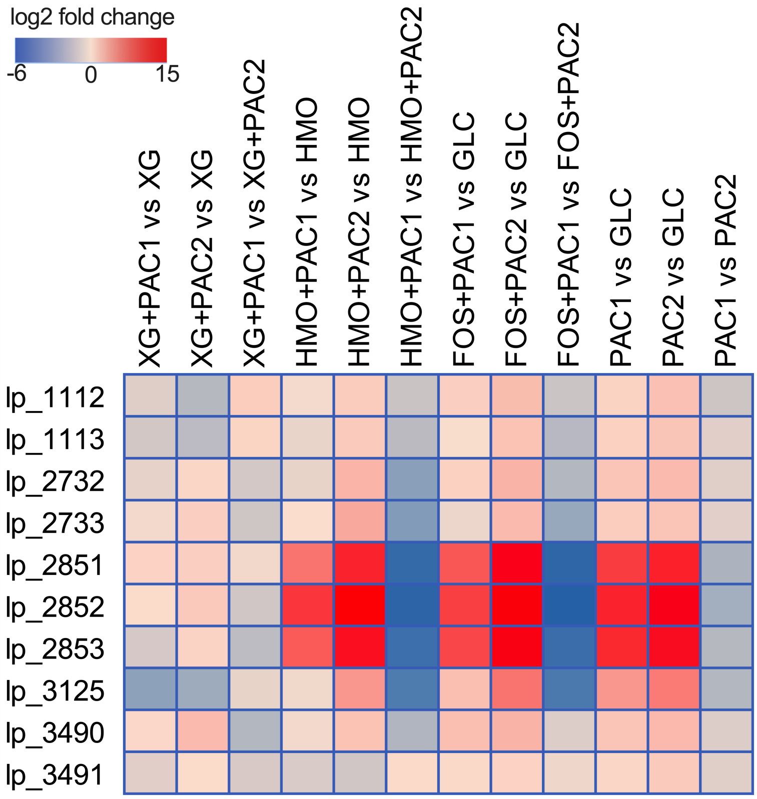

Fig. S12 Relative gene expression of predicted fumarate reductases within the global transcriptome. The log_2 fold change gene expression of potential fumarate reductases involved polyphenol metabolism. The heatmap was generated from independent biological duplicates from raw reads using the R package DESeq2.

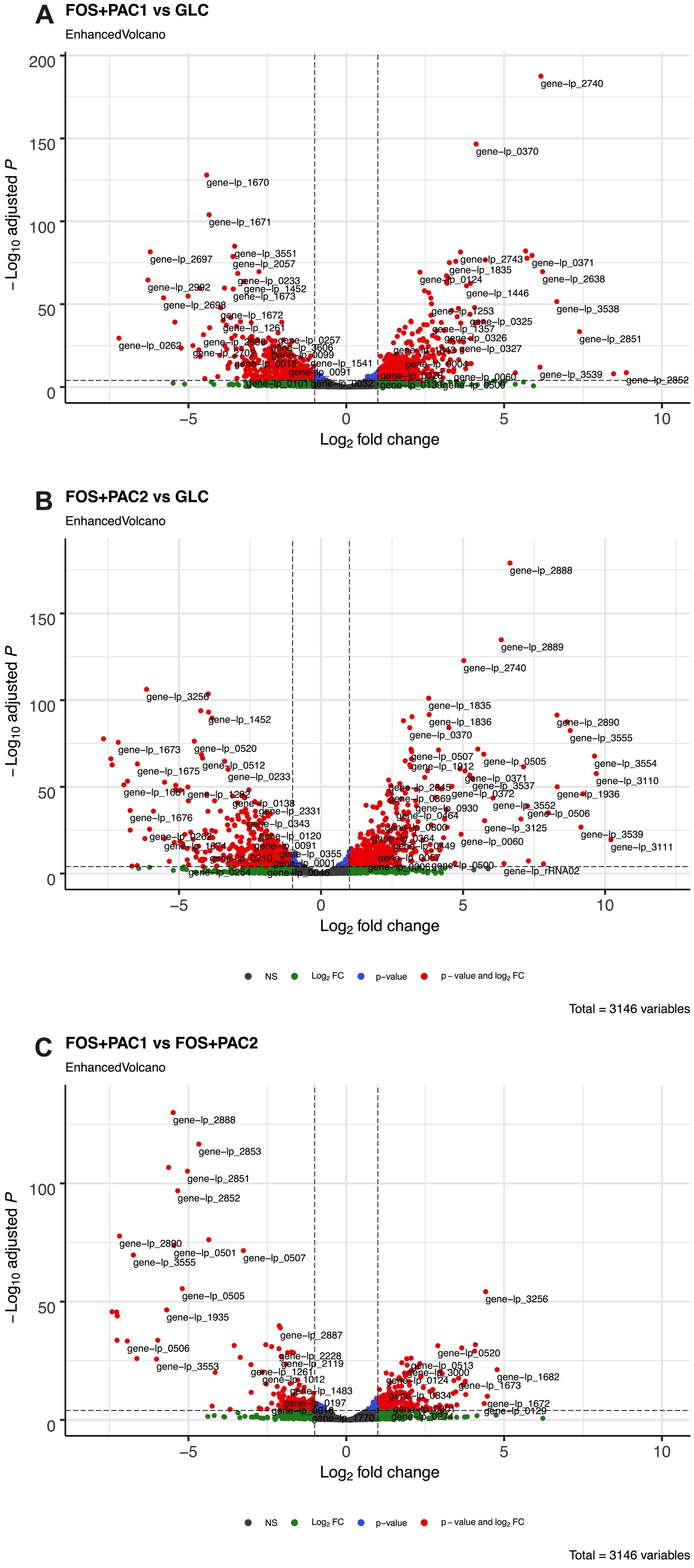

Fig S13 Volcano plots of differential gene expression profiles of pairwise comparisons. Red dots represent upregulated and downregulated differentially expressed genes by FOS with PAC1 (A), FOS with PAC2 (B) compared to glucose (GLC), FOS with PAC1 compared to FOS+PAC2 (C). The x axis represents log2 fold change of gene expression and the y axis represents the –log10 adjusted p value (FDR) calculated from R package DESeq2.
